# 2D Black Phosphorus: from Preparation to Applications for Electrochemical Energy Storage

**DOI:** 10.1002/advs.201700491

**Published:** 2018-02-23

**Authors:** Shuxing Wu, Kwan San Hui, Kwun Nam Hui

**Affiliations:** ^1^ Institute of Applied Physics and Materials Engineering University of Macau Avenida da Universidade Taipa Macau China; ^2^ School of Mathematics University of East Anglia Norwich NR4 7TJ UK

**Keywords:** 2D black phosphorus, energy storage, instability, passivation, preparation methods

## Abstract

Black phosphorus (BP) is rediscovered as a 2D layered material. Since its first isolation in 2014, 2D BP has triggered tremendous interest in the fields of condensed matter physics, chemistry, and materials science. Given its unique puckered monolayer geometry, 2D BP displays many unprecedented properties and is being explored for use in numerous applications. The flexibility, large surface area, and good electric conductivity of 2D BP make it a promising electrode material for electrochemical energy storage devices (EESDs). Here, the experimental and theoretical progress of 2D BP is presented on the basis of its preparation methods. The structural and physiochemical properties, air instability, passivation, and EESD applications of 2D BP are discussed systemically. Specifically, the latest research findings on utilizing 2D BP in EESDs, such as lithium‐ion batteries, supercapacitors, and emerging technologies (lithium–sulfur batteries, magnesium‐ion batteries, and sodium‐ion batteries), are summarized. On the basis of the current progress, a few personal perspectives on the existing challenges and future research directions in this developing field are provided.

## 1 Introduction

Since Novoselov et al. exfoliated graphene from graphite via the mechanical cleavage method in 2004, 2D materials have attracted intensive interest.^1^, ^2^, ^3^ Graphene is a 2D single layer of sp^2^‐bonded carbon atoms that are densely packed in a honeycomb crystal lattice that has a series of unexpected chemical and physical features, such as remarkably high electron mobility at room temperature (15 000 cm^2^ V^−1^ s^−1^),^4^ strong mechanical strength (≈1 TPa),^5^ excellent optical transparency (≈97.7%),^6^ intriguing thermal conductivity (4.84 × 10^3^–5.30 × 10^3^ W m^−1^ K^−1^),^7^ and large theoretical specific surface area (SSA ≈ 2630 m^2^ g^−1^).^8^ Numerous laboratory results demonstrate the potential of graphene in transforming the landscape of current electrochemical energy storage devices (EESDs).^9^, ^10^ The unprecedented properties of graphene have led to massive research efforts on other 2D materials for this application, such as transition‐metal oxides/hydroxides,^11^, ^12^, ^13^, ^14^, ^15^ transition‐metal dichalcogenides (TMDs),^2^, ^16^, ^17^ hexagonal boron nitride (h‐BN),^18^, ^19^ and transition‐metal carbides and nitrides (MXenes).^20^, ^21^, ^22^, ^23^ In a 2D material, the atomic organization and bond strength along the two dimensions are analogous and significantly stronger than those in a third dimension.^23^, ^24^ Its physicochemical characteristics are different from those of the bulk counterpart, and two well‐established allotropes, namely, single layer and few layers, are involved.^16^, ^25^ The following striking properties make the 2D material a predominantly promising material for EESDs: (1) its large lateral size and ultrathin characteristic endow it with ultrahigh SSA and high ratios of exposed surface atoms, thereby making it an ideal platform for energy storage;^26^ (2) its “all‐surface” nature offers an opportunity to engineer properties tailored by surface treatments;^16^ (3) it can intercalate ions and store energy in the 2D channels among nanosheets through the rapid ion adsorption mechanism;^11^, ^27^ (4) it can serve as a building block for various hybrid and hierarchical nanostructures from zero dimension to three dimensions, because no single material can perfectly fulfill the rigorous requirements of EESDs;^28^ and (5) its atomic thickness offers maximum mechanical flexibility and high packing density, thereby making it promising for developing highly flexible EESDs.^26^, ^29^

**Figure 1:**
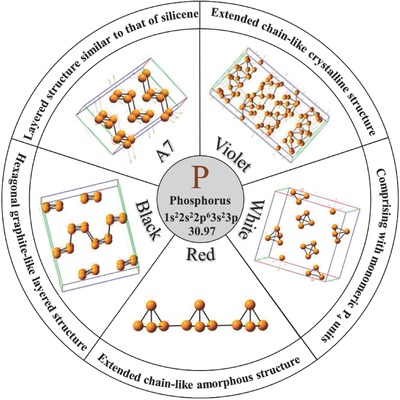
Allotropic forms of phosphorus. Reproduced with permission.^33^ Copyright 2017, American Chemical Society.

The family of 2D materials has recently been augmented by 2D black phosphorus (BP, mono‐ or/and few‐layer BP). Phosphorus is among the abundant elements on Earth, making up ≈0.1% of the Earth's crust.^30^, ^31^ Phosphorus exists in various allotropes, including white phosphorus, red phosphorus, BP, violet phosphorus, and A7 phase.^32^, ^33^ **Figure** **1** presents the various allotropic forms of phosphorus.^33^

**Figure 2:**
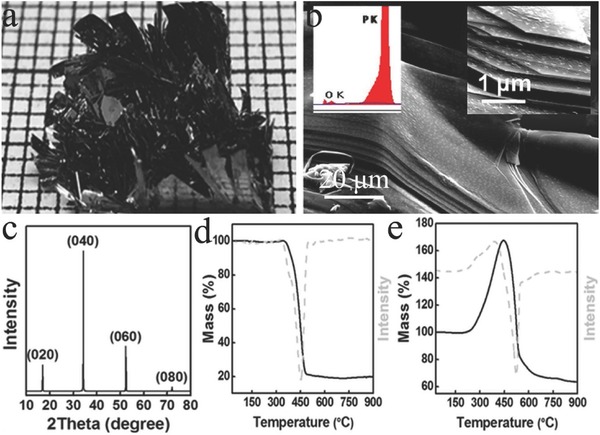
a) Optical image of BP. Reproduced with permission.^36^ b) Scanning electron microscopy (SEM) image of the layered structure of BP. Inset: energy dispersive X‐ray spectroscopy (EDX) spectra of BP crystal (left) and magnified SEM image revealing the presence of sharp edges (right). Reproduced with permission.^35^ Copyright 2016, Nature Publishing Group. c) XRD pattern of the BP. TG–DTG curves of BP under d) nitrogen flow and e) air flow. Reproduced with permission.^36^

BP is the most thermodynamically stable phosphorus allotrope under ambient conditions. **Figure** **2**a shows the optical image of the BP crystal.^34^ BP atoms are strongly bonded in plane, thereby forming layers, and individually layered atoms are stacked together by weak van der Waals forces. Figure 2b shows the layered structure of BP.^35^ The X‐ray diffraction (XRD) pattern (Figure 2c) of BP reveals four peaks that are indexed to the (020), (040), (060), and (080) planes in the 2θ range of 10°–70°.^36^ A comparison of two samples under different ambient atmospheres (nitrogen an air flow) shows that BP is relatively stable with inert gases, which is demonstrated by using thermogravimetry and differential thermogravimetry (TG–DTG) (Figure 2d,e).^36^ No sign of thermal decomposition is observed until 210 °C in air (Figure 2e).^36^

**Figure 3:**
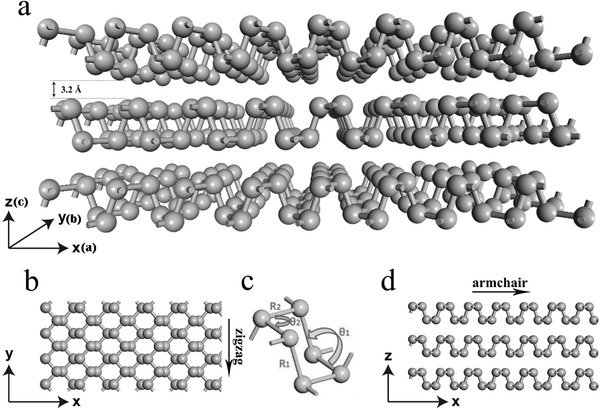
Crystal structure of 2D BP: a) perspective side view; b) top view; c) structure parameters (*R*_1_, *R*_2_) and angles (θ_1_, θ_2_). Reproduced with permission.^40^ Copyright 2016, Taylor & Francis Online Publishing; and d) side view. Reproduced with permission.^37^ Copyright 2017, The Royal Society of Chemistry.

The individual layers of bulk BP can be mechanically exfoliated down to monolayers, similar to graphene from graphite. At normal conditions, the bulk BP structure is orthorhombic with space group *Cmca*. The crystal structure is shown in **Figure** **3**a.^37^ Each phosphorus atom is bonded to three neighboring atoms through sp^3^‐hybridized orbitals, thereby making the atoms resemble a puckered honeycomb structure. In addition, each atom has a lone electron pair, and the remaining lone pairs make phosphorus reactive to air.^38^ The unit cell consists of two layers and is side‐centered orthorhombic, with lattice constants *a* = 4.47 Å and *b* = 3.34 Å.^39^ Each puckered layer can be viewed as two rows of parallel atomic planes in which the phosphorus atoms on each plane form zigzag (ZZ)‐like (Figure 3b) and armchair (AC)‐like (Figure 3d) geometries along the *y‐* and *x*‐directions, respectively.^37^ BP has a mirror reflection symmetry only in the *y*‐direction. The P—P distance of the connection between top and bottom atoms (*R*_1_) is long (2.28 Å), and the corresponding angle (θ_1_) is 102.42° (Figure 3c). The bond angle along the ZZ direction (θ_2_) is 96.16°, and the bond length (*R*_2_) with the nearest atoms is 2.25 Å.

Bulk BP is a direct band gap p‐type semiconductor with good electrical conductivity (≈10^2^ S m^−1^), reasonable density (2.69 g cm^−3^), and an intrinsic energy gap of ≈0.34 eV.^41^ This semiconductor also exhibits great electrical properties with electron and hole mobilities of 220 and 350 cm^2^ V^−1^ s^−1^, respectively.^42^ BP has three crystalline phases, namely, orthorhombic, rhombohedral, and simple cubic phases.^30^ Under high pressure, semiconducting orthorhombic BP, which is stable under ambient conditions, can transform to a semimetallic rhombohedral structure at 5.5 GPa at room temperature.^43^ BP has two types of bonds, namely, covalent intralayer bonding and weak interlayer van der Waals bonding.^44^ With increasing pressures, the distances among the individual layers decrease faster than the intralayer atomic separations because the interlayer van der Waals coupling is relatively weaker.^45^ Under a much higher pressure (10 GPa), the semimetallic rhombohedral structure converts further into metallic cubic phase because of an internal distortion.^46^ Bulk BP was first synthesized by Bridgman in 1914 under high pressure (1.2 GPa) at 200 °C.^47^ The conversion from white phosphorus to bulk BP took 5–30 min. Bulk BP was also prepared from white and red phosphorus at room temperature at 3.4 and 8.5 GPa, respectively.^48^, ^49^ Without using high pressure, bulk BP was prepared using mercury as a catalyst^50^ and the bismuth‐flux,^51^ mineralizer‐assisted short‐way transport reaction,^52^, ^53^ and sonochemistry methods.^44^

**Figure 4:**
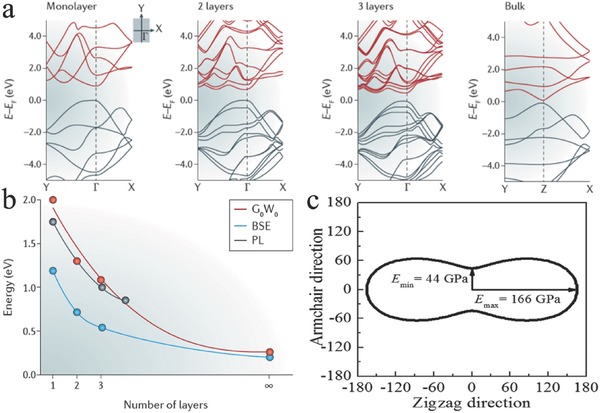
a) Band structures for the monolayer, bilayer, and trilayer of BP and bulk BP. b) Band gap variation as a function of the number of layers, the theory (*G*_0_ *W*_0_ and BSE) and the experiment (PL). Reproduced with permission.^57^ Copyright 2016, Macmillan Publishers Limited. c) Directional dependence of the Young's modulus of a monolayer BP. Reproduced with permission.^59^ Copyright 2014, AIP Publishing LLC.

Although bulk BP was discovered more than a century ago, a few studies have focused on the use of BP because of its difficult synthesis process, which includes high‐temperature and high‐pressure conditions. Until 2014, the monolayer of BP (also termed “phosphorene”) was isolated successfully by using the same sticky‐tape technique as for graphene.^54^, ^55^ The lattice constants for the monolayer of BP are *a* = 4.58 Å and *b* = 3.32 Å. The band gap of BP is layer dependent, and monolayer BP has a direct band gap of ≈2.0 eV at the Γ point of the first Brillouin zone.^54^ The shapes of the band gaps of BP with monolayer and multilayers are similar in that they have the same translational structural symmetry and bond interactions (**Figure** **4**a).^56^, ^57^ The band gap remains direct at the Γ point of the Brillouin zone. A disparity exists only near the Fermi level and close to the Γ points where the energy gap has a different opening. Figure 4b shows the band gap as a function of thickness obtained using theoretical (*G*_0_ *W*_0_ and Bethe–Salpeter equation (BSE)) and experimental (photoluminescence (PL)) methods. Considering the puckered structure, 2D BP has a much larger SSA than graphene, which has a plane structure.^58^ However, no report on the SSA for 2D BP in theoretical prediction and experimental measurements has been provided. Moreover, 2D BP demonstrates superior mechanical flexibility, which is a promising alternative for flexible devices. Theoretical studies have shown that the Young's modulus of 2D BP (≈94 GPa) is direction dependent (166 and 44 GPa along the ZZ and AC directions, respectively), as shown in Figure 4c.^59^ **Table** **1** summarizes the structures and properties of monolayer BP and bulk BP in comparison with the more mature and well‐studied graphene. On the basis of intrinsic characteristics, 2D BP has been investigated for use in various applications, including transistor,^54^, ^60^, ^61^, ^62^ solar cell,^63^, ^64^ thermoelectric,^65^, ^66^, ^67^ heterojunction p–n diode,^68^, ^69^ sensing,^70^, ^71^ photovoltaic devices,^72^ and photodetectors.^73^, ^74^

**Table 1:**
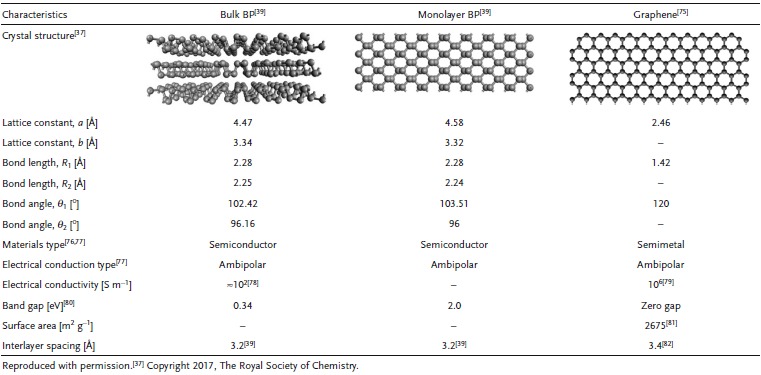
Summary of structure and properties of monolayer BP and bulk BP compared to those of graphene

**Figure 5:**
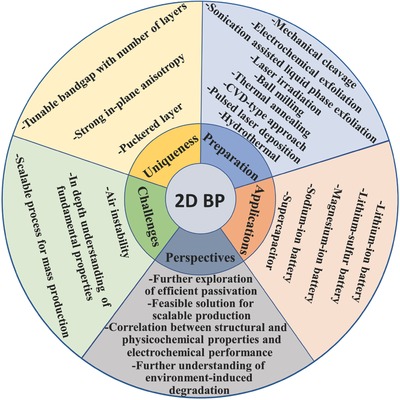
Graphical summary of the present review.

The unique structure and properties of 2D BP have attracted significant attention in the field of EESDs, and notable progress in this field has been achieved. The specific merits of BP from graphene and other 2D materials such as direct band gap (from −0.3 to 2.0 eV) which is highly tunable with number of layers, strong in‐plane anisotropy, and puckered layer. Although a few reviews on 2D BP have been published,^30^, ^38^, ^56^, ^57^, ^77^, ^80^, ^83^, ^84^, ^85^, ^86^, ^87^, ^88^, ^89^, ^90^ previous sporadic studies on 2D BP in energy storage systems have not been systematically presented. Thus, a comprehensive overview on the state of 2D BP research for EESDs is significant. This report is organized as follows: we first review the recent experimental and theoretical progress in 2D BP preparation and the structural and physiochemical properties, air instability, and passivation of 2D BP. We then focus on the latest advances in the use of 2D BP in lithium‐ion batteries (LIBs), lithium–sulfur batteries (LSBs), magnesium‐ion batteries (MIBs), sodium‐ion batteries (SIBs), and supercapacitors (SCs). Finally, we discuss the current challenges in preparing 2D BP and its application in EESD, and present an outlook to highlight future research directions. The overall summary of topics considered for 2D BP is presented in **Figure** **5**.

## 2 Preparation

**Figure 6:**
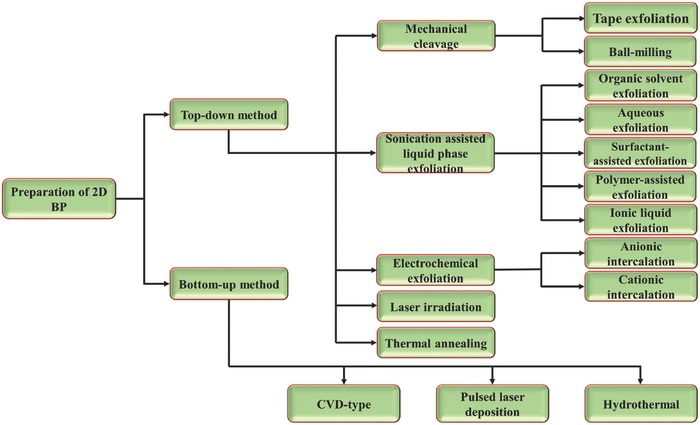
Overview of currently available preparation strategies for fabricating 2D BP.

The reliable production of 2D BP with uniform size is significant for exploring its structural and physiochemical properties and its potential applications. Driven by the interesting properties and promising applications of 2D BP, concerted research efforts have been dedicated to developing various synthetic strategies for fabricating 2D BP. Reliable preparation methods, such as mechanical cleavage, liquid exfoliation, and chemical synthesis, have been explored to produce 2D BP for fundamental and applied research. All methods can generally be divided into top‐down and bottom‐up approaches. The top‐down method typically uses mechanical force or chemical intercalation to break the weak van der Waals bonding among stacked layers to obtain mono‐ or few‐layer nanosheets from bulk BP. The bottom‐up approach relies on the direct synthesis of 2D BP from different molecule precursors via chemical reactions. In this review, we summarize the current methods used for fabrication along with highlights of their advantages and disadvantages (**Figure** **6**).

### 2.1 Top‐Down Methods

#### 2.1.1 Mechanical Cleavage

The mechanical cleavage approach is a traditional method for exfoliating layered bulk materials to achieve 2D flakes. In 1990, Seibert et al. prepared thin, highly oriented pyrolytic graphite films by optically peeling thin layers from a bulk sample with transparent tape.^91^ Similarly, Novoselov et al. prepared single‐layer graphene using Scotch tape in 2004.^1^ The energy required to exfoliate layered crystals can be quantified by the surface energy and is the energy that is needed to remove a single layer from the crystal divided by twice the single‐layer surface area.^92^

**Figure 7:**
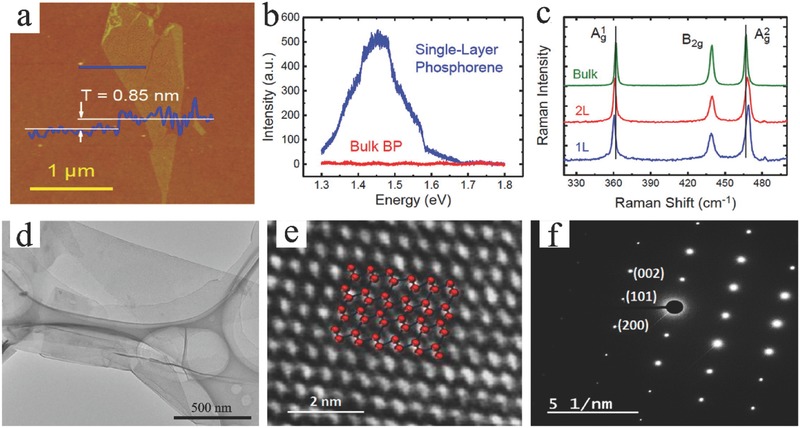
a) AFM image of a monolayer BP crystal with a measured thickness of ≈0.85 nm. b) Photoluminescence spectra for monolayer BP and bulk BP samples on a 300 nm SiO_2_/Si substrate. c) Raman spectra of monolayer, bilayer, and bulk BP. Reproduced with permission.^55^ Copyright 2014, American Chemical Society. d) TEM image of few‐layer 2D BP. Reproduced with permission.^95^ Copyright 2014, Springer. e) High‐resolution TEM image and f) SAED pattern for few‐layer 2D BP. Reproduced with permission.^94^ Copyright 2015, American Chemical Society.

Mechanical cleavage using adhesive tape can be applied to peel 2D BP flakes from their bulk BP. According to multilevel quantum‐chemical calculations, the exfoliation energy of BP is −151 meV per atom.^93^ In a typical exfoliation process,^55^ bulk BP is first adhered onto a piece of Scotch tape, and another piece of tape is then attached onto another surface. Subsequently, one of the Scotch tapes is detached from the bulk BP. This process is repeated several times to acquire 2D BP flakes. Thereafter, the Scotch tape with the thin BP flakes is attached onto the Si/SiO_2_ substrate. Finally, all samples are cleaned with acetone, methanol, and isopropyl alcohol (IPA) to remove the Scotch tape residue. The solvent residue is removed under a 180 °C postbake process. The thickness of the monolayer BP is ≈0.85 nm, which is larger than the theoretical value of 0.6 nm (**Figure** **7**a).^55^ The PL of exfoliated monolayer BP is observed in visible wavelengths, whereas no PL signal is observed within the detection spectrum range for bulk BP (Figure 7b). The Raman spectra of 2D BP also exhibit thickness dependence. The $A_{g}^{1}$ and $A_{g}^{2}$ modes shift toward each other when the thickness is increased (Figure 7c). Plasma is used to treat the BP flakes after exfoliation to thin them.^94^, ^95^ Figure 7d shows a typical transmission electron microscopy (TEM) image that presents the morphology of the few‐layer 2D BP. Figure 7e shows a high‐resolution TEM image of 2D BP. Perfect and orderly atomic arrangements are clearly shown. The selective area energy diffraction (SAED) pattern suggests high crystallinity (Figure 7f). As a result of the low production yield, the Si/SiO_2_ substrate surface is active with O_2_ plasma to enhance the yield before exfoliation.^96^ Meanwhile, modified tape exfoliation techniques are developed. Bulk BP is first exfoliated to polydimethylsiloxane stamp,^97^ viscoelastic stamp,^98^ or poly(methyl methacrylate)/poly(vinyl alcohol) stack,^99^ and then transferred onto another substrate (such as Si/SiO_2_ and glass frame).^100^ Given that this process relies only on applied shear force and no chemical reactions occur, the obtained 2D BP flakes have the same crystal structures as their bulk BP crystals. Thus, mechanical cleavage produces high‐quality 2D BP flakes that are suitable for fundamental studies and for fabricating high‐performance devices. Although this method is relatively simple, fast, and cost effective, it has the following limitations: (1) the production yield is low, and the method is impractical for large‐scale applications; (2) a substrate is always required to support the exfoliated 2D flakes; and (3) the method lacks systematic control of the thickness, size, and shape of 2D flakes. The mechanical cleavage method is seldom used in applications in EESDs.

Recently, Zhu et al. successfully fabricated a large‐scale 2D BP through a facile solid‐state mechanochemical cleavage method by ball milling with the addition of LiOH, which can be a promising method for practical applications.^31^ With the addition of LiOH, the edge of the 2D BP nanosheets can be further functionalized by hydroxyl groups during the high‐energy mechanical ball milling process, resulting in the efficient mechanochemical cleavage of the P—P bonds.

#### 2.1.2 Sonication‐Assisted Liquid‐Phase Exfoliation

**Figure 8:**
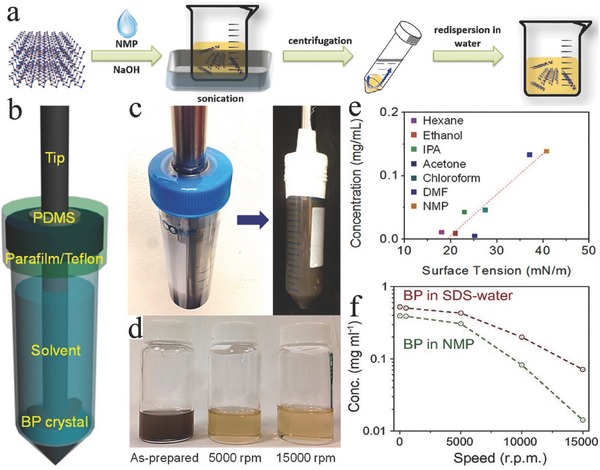
a) Schematic representation of the synthesis process of basic‐NMP‐exfoliated 2D BP. Reproduced with permission.^101^ b) Schematic and c) photograph of the custom‐tip ultrasonication setup for BP exfoliation. d) Photograph of a BP dispersion in NMP after different centrifugation speeds. e) 2D BP concentration plot for various solvents with different surface tensions after 5000 rpm centrifugation. Reproduced with permission.^106^ Copyright 2015, American Chemical Society. f) Concentration of the 2D BP dispersion in SDS water and NMP after different centrifugations. Reproduced with permission.^124^ Copyright 2016, National Academy of Sciences.

Sonication‐assisted liquid‐phase exfoliation is a reliable method for producing 2D BP at bulk scale, which is suitable for EESDs. The exfoliation process generally consists of three steps: (1) immersion into solvent, (2) ultrasonication, and (3) purification (**Figure** **8**a).^83^, ^101^ The selection of the optimum solvent plays a key role in utilizing the full potential of this technique. Optical absorption spectroscopy proves that solvent, which has a surface tension of 35–40 mJ m^−2^, maximizes the exfoliation rate.^16^ The utilization of solvent that has a surface tension similar to the surface energy of a 2D material is critical to minimizing the energy cost of exfoliation and prohibiting the restacking of nanosheets.^102^ Bulk BP has been successfully exfoliated using several solvents. Organic solvents, including *N*‐methyl‐2‐pyrrolidone (NMP),^101^, ^103^, ^104^, ^105^, ^106^, ^107^ *N*‐cyclohexyl‐2‐pyrrolidone (CHP),^108^ dimethylformamide (DMF),^109^, ^110^ dimethyl sulfoxide,^110^, ^111^ and acetone,^112^, ^113^ have been used to prepare uniform and stable dispersions. Among these solvents, NMP (40 mJ m^−2^) and DMF (37.1 mJ m^−2^) have surface energy values that are close to the exfoliation criterion of 2D BP (35–40 mJ m^−2^).^114^ Kang et al.^106^ investigated seven kinds of organic solvents, namely, acetone, chloroform, hexane, ethanol, IPA, DMF, and NMP, for BP exfoliation. During BP exfoliation, exfoliation reaction was performed in a dark Ar glove box with the use of a modified sealed‐tip ultrasonicator setup (Figure 8b,c). The concentrations of the BP dispersions were separated and collected according to different centrifugation speeds from brown (as‐prepared) to light yellow color (5000 rpm) and pale yellow color (15 000 rpm) (Figure 8d). Results showed that the BP concentration increased with the surface tension of the solvent, and NMP was the optimal solvent for BP exfoliation (Figure 8e). In another research, 18 solvents were explored, and benzonitrile produced the highest concentration (0.11 mg mL^−1^).^115^

Although organic solvent exfoliation enables the large‐scale exfoliation of BP and attainment of uniform BP dispersion, the production yield of this method is generally low, and as‐exfoliated BP is not stable in common solvent. Accordingly, modified methods have been developed to exfoliate 2D BP with the preferred number of layers (thickness), high quality, and high yield. Guo et al.^101^ reported a high‐yield production of BP using a basic‐NMP solvent exfoliation method with the addition of NaOH. The exfoliated BP also showed excellent stability, which is attributed to the negative charge of BP due to the OH^−^ ions absorbed on the surface of BP. Xu et al.^109^ reported a fast BP exfoliation approach using a small molecule with a DMF solvent. In this small molecule‐assisted liquid‐phase exfoliation method, bulk BP is dissolved in DMF with the simultaneous addition of phytic acid. As a result of the unique phytic acid structure with rich polar phosphorus and hydroxyl functional groups, phytic acid molecules easily interacted with BP, thereby enhancing the exfoliation rate. Recently, Choi et al. reported an acoustic‐microfluidic process, which achieved 45% exfoliation efficiency in 6 min.^116^

Organic solvents can efficiently exfoliate bulk BP, but their adsorption on the surface of as‐exfoliated nanosheets is too intimate to be removed and will adversely affect the intrinsic properties of BP in applications.^117^ Therefore, aqueous solvent was explored and developed for BP exfoliation. Studies have shown that a 2D BP with clean surface can be obtained by directly exfoliating bulk BP in water.^117^, ^118^, ^119^ Normally, surfactants are added to water to tune the water surface energy because the surface energy of water (72.75 mJ m^−2^) is higher than the criterion (35–40 mJ m^−2^).^120^ The surfactant‐assisted exfoliation of BP in water was first reported by Kang et al.^121^ Deoxygenated water with 2% (w/v) sodium dodecyl sulfate (SDS) was used as solvent. Their results showed that BP dispersion prepared with SDS in aqueous solution produces a stable solution, while BP dispersed without SDS precipitates quickly. Their results also showed that the 2D BP dispersion was stabilized with the incorporation of SDS, and a higher concentration of BP sheets can be achieved in water with surfactant SDS than in water with NMP solvent (Figure 8f). The 2D BP dispersions were stabilized only in aqueous solutions with the incorporation of amphiphilic surfactants. For example, Triton X‐100 (TX‐100) is employed to assist exfoliation in water and plays a pivotal role in retarding the oxidative degradation process.^122^, ^123^

Recently, the exfoliation of bulk BP has been demonstrated in water using polymer. Polyvinylpyrrolidone (PVP)‐assisted exfoliation was developed by Peng et al., and the thickness of the as‐fabricated 2D BP was 2–3 nm.^124^ The selection of a suitable polymer–solvent combination is key to obtaining 2D BP dispersion with high concentration in polymer‐assisted exfoliation. In the case of graphite exfoliation, May et al.^125^ predicted that maximal graphene concentration can be achieved when the polymer and solvent have similar Hildebrand solubility parameters as graphene sheets, which could be a reference for BP exfoliation.^114^

As popular green solvents, ionic liquids (ILs) have been explored and used in bulk BP exfoliation. Lee et al. reported the liquid‐phase exfoliation of bulk BP with the incorporation of two representative ILs ([Emim][Tf2N] and [Bmim][Tf2N]).^126^ The strong van der Waals interaction between the BP nanosheets and [Bmim][Tf2N]) leads to a more effective exfoliation of bulk BP than [Emim][Tf2N] because [Bmim][Tf2N] has longer alkyl groups. In a recent paper by Mu et al., nine different ILs were successfully demonstrated to achieve exfoliated BP nanoflakes, and the as‐prepared solution can be stabilized without any obvious aggregation for months.^127^ A high concentration (up to 0.95 mg mL^−1^) of 2D BP was obtained in 1‐hydroxyethyl‐3‐methylimidazolium trifluoromethansulfonate ([HOEMIM]‐[Tfo]) IL, which was the highest reported concentration of 2D BP dispersions to date.

**Figure 9:**
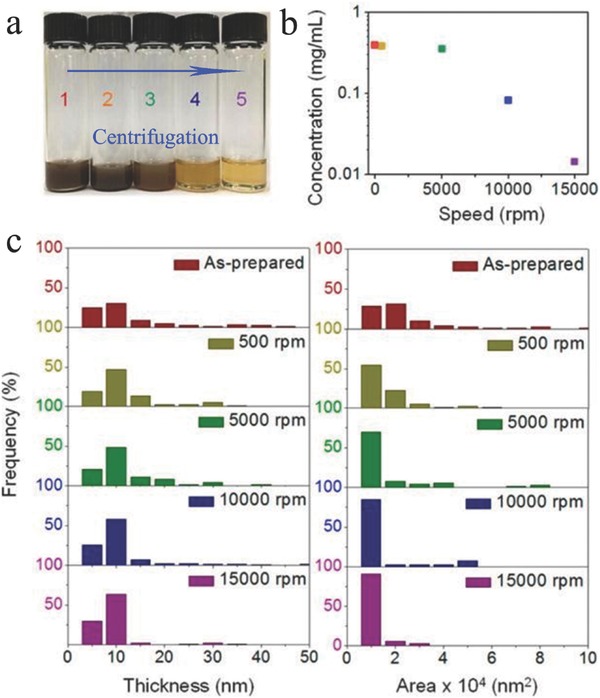
Concentration, flake thickness, and lateral area distribution of exfoliated BP in NMP with different centrifugation speeds (1: as‐prepared; 2: 500 rpm; 3: 5000 rpm; 4: 10 000 rpm; 5: 15 000 rpm). Reproduced with permission.^106^ Copyright 2015, American Chemical Society.

Generally, ultrasonication can effectively break the interlayer van der Waals bonding in the exfoliation process but not the covalent intralayer bonding. Studies have shown that ultrasonic waves create cavitation bubbles that collapse into high‐energy jets via pressure release, which breaks up the layered crystallites and generates exfoliated nanoflakes.^128^ As ultrasonication time is one of the key factors that dictate the quality of the resultant dispersion, the exfoliation efficiency and concentration of 2D BP can be increased by extending the ultrasonication time, but it can also reduce the lateral dimensions. According to Lewis and co‐workers, the reduction in lateral dimensions was obvious when the ultrasonication increased from 24 to 48 h.^103^ After ultrasonication, the resultant solution can be purified by the centrifugation process. The centrifugation rate is another critical parameter for yielding high‐quality 2D nanoflakes. **Figure** **9**a shows a photograph of exfoliated 2D BP solutions centrifuged at different speeds. The color of dispersed 2D BP solution changed from dark brown to light yellow as higher centrifugation speeds were used. BP concentration (Figure 9b), flake thickness, and lateral size (Figure 9c) decreased with increasing centrifugation. Generally, the resultant suspension is centrifuged at a low rate, such as 1500 rpm, to remove the residual unexfoliated particles and centrifuged at a rate higher than 10 000 rpm to separate relatively thick nanoflakes. Despite massive production using this method, the yield of monolayer nanoflakes obtained by this method is relatively low, and the lateral dimensions of the obtained nanoflakes are small. Other deficiencies, such as ultralong sonication time and uncontrollable thicknesses, are other obstacles to the development of 2D BP‐based materials.

#### 2.1.3 Electrochemical Exfoliation

**Figure 10:**
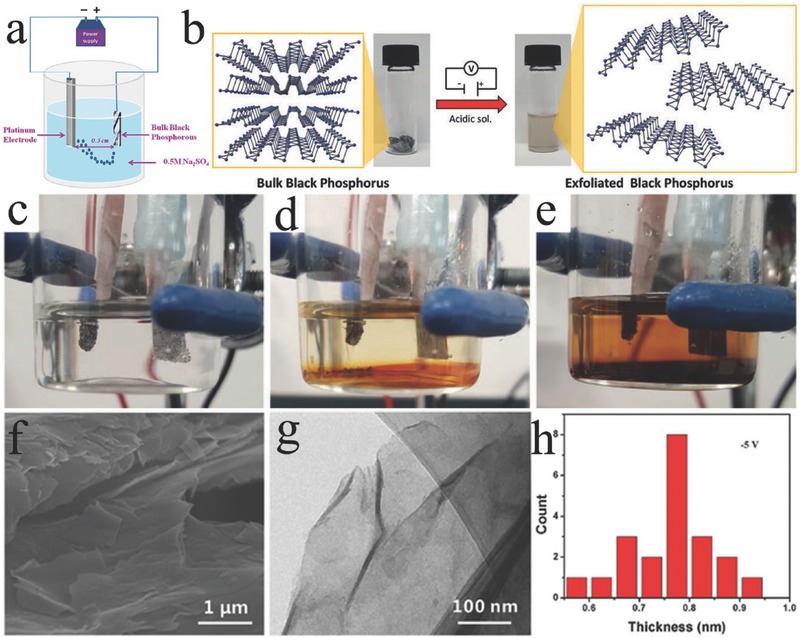
a) Experimental setup used for the electrochemical exfoliation of BP nanosheets. Reproduced with permission.^132^ Copyright 2016, American Chemical Society. b) Exfoliation procedure. The BP crystal is exfoliated in an acidic aqueous solution with the application of a DC voltage. The starting BP crystals (left) and the exfoliated BP dispersion in DMF (right) are also presented. c) No potential applied; d) 20 min after applying a voltage; and e) after 2 h of applied voltage. Reproduced with permission.^135^ f) SEM, g) TEM, and h) the corresponding statistics of 2D BP obtained. Reproduced with permission.^134^

Electrochemical exfoliation (e.g., anodic oxidation and cationic intercalation) has been demonstrated as a more facile, faster, and more environmentally friendly approach to prepare high‐quality 2D nanoflakes at a large scale compared with liquid‐phase exfoliation.^129^ In the working mechanism of the electrochemical exfoliation of BP, when a voltage is applied to bulk BP as the working electrode in a conductive solution (electrolyte), the generated electrical current courses through the structural deformation of the layered BP material and yields 2D BP nanoflakes.^130^, ^131^ The success of exfoliation relies highly on the selection of electrolyte, operating voltage, and precursor.^130^ To date, anionic and cationic intercalations have been successfully applied in the exfoliation of bulk BP in electrochemical exfoliation. For example, a two‐electrode system electrochemical exfoliation in a 0.5 m Na_2_SO_4_ aqueous solution was developed by Erande et al., where bulk BP and Pt wire were used as the anode and cathode, respectively (**Figure** **10**a).^132^ The exfoliated BP nanoflakes possessed 3–15 stacked layers, and the lateral dimension was 0.5–30 µm. In a recent report, Ambrosi and co‐workers,^133^ using bulk BP as anode, Pt foil as cathode, and 0.5 m H_2_SO_4_ as electrolyte, established a two‐electrode system to exfoliate bulk BP into nanosheets (Figure 10b–e). Few‐layer‐thick BP nanosheets were produced. The exfoliation efficiency and quality of the produced BP nanosheets could be controlled by changing the anodic potential to the BP crystal. The anodic oxidation for the electrochemical exfoliation can lead to the partial oxidization of a 2D material. Cationic insertions without oxidizing conditions can be an alternative methodology for overcoming the oxidation of 2D material. Huang et al.^134^ proposed a layer‐tunable cathodic exfoliation method through controlling the intercalation rate of tetraalkylammonium cations to prepare 2D BP. Bulk BP was used as cathode, and Pt sheet was used as anode. Large‐area sheets, the sizes of which reached dozens of micrometers, were obtained at the potential of −5 V (Figure 10f). Ultrathin slices of 2D BP were clearly observed (Figure 10g). The thickness of the as‐prepared 2D BP was mainly distributed between 0.76 and 0.79 nm, which correspond to approximately two layers of BP (Figure 10h), and the layer number of the as‐prepared 2D BP (from 2 layers to 11 layers) can be controlled by changing the applied potential.

#### 2.1.4 Other Top‐Down Methods

Alternatively, Suryawanshi et al. synthesized 2D few‐layered BP nanosheets via a one‐step facile laser irradiation technique.^136^ In their study, the laser irradiation of bulk BP in isopropyl alcohol resulted in the formation of micrometer‐sized 2D BP sheets. During the laser exfoliation process, laser illumination generates thermal shock, which leads to cracking and BP flake detachments from the bulk BP. Recently, Fan et al. demonstrated a two‐step thermal annealing method under the sequential flow of air and N_2_/H_2_ mixture gases to thin down BP to achieve a seven‐layer BP flake under 340 °C within 2 min, which can be potentially applied for mass production.^137^ Significantly, the thickness of the BP flakes can be controlled by adjusting the annealing temperature.

### 2.2 Bottom‐Up Methods

Over the decades, chemical vapor deposition (CVD) has been widely used as a materials processing technology and regarded as one of the most promising bottom‐up methods. CVD is a process that involves the use of volatile precursors at high temperature, in which the volatile precursors react and/or decompose on the substrate surface to produce 2D nanomaterials. To date, the CVD method has been successfully used to fabricate many 2D nanomaterials, including graphene, TMDs, h‐BN, and metal oxides.^25^, ^27^, ^29^, ^138^ However, investigations on the CVD growth of 2D BP are limited. Recently, Smith et al.^139^ demonstrated an in situ CVD‐type approach for growing crystalline 2D BP from red phosphorus directly on the silicon substrate. The as‐fabricated 2D BP with average areas >3 µm^2^ had approximately four layers. Yang et al.^60^ demonstrated the success of the fabrication of BP ultrathin films grown on graphene/copper or SiO_2_/Si substrates by using pulsed laser deposition technology. However, the BP grown by the pulsed laser deposition method has a highly disordered amorphous structure.

**Figure 11:**
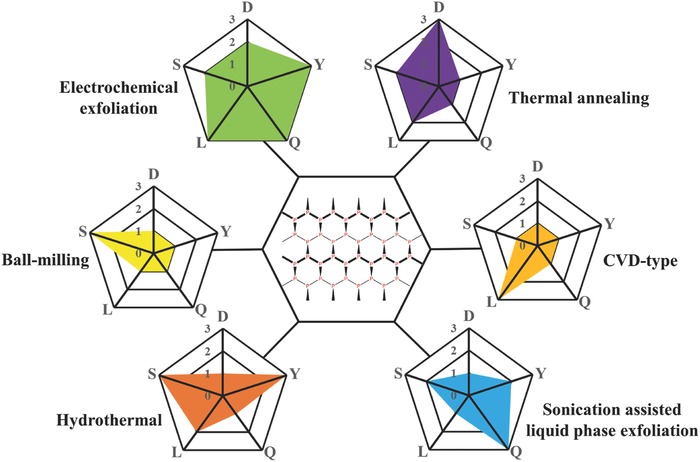
Comparison of six 2D BP fabrication methods. Each method was evaluated in terms of duration (D), yield (Y), 2D BP quality (Q), lateral dimension (L), and scalability (S) of the overall fabrication process. For D, 1 = high, 2 = moderate, and 3 = low. For Y, Q, L, and S, 1 = low, 2 = moderate, and 3 = high.

To date, direct chemical growth and chemical synthesis are a major unexplored area for synthesizing 2D BP. They show great potential for the scalable production of 2D BP toward practical EESD applications. Zhao et al.^140^ recently reported a novel mild‐phase transition for preparing BP nanosheets. Phase transformation and the Gibbs free theory indicate that the generation of BP from red phosphorus is a spontaneous process (Δ*G* = −16.37 kJ mol^−1^ < 0).^47^ In their study, red phosphorus microspheres were selected as raw material, and NH_4_F was utilized to reduce the surface activation energy of the raw material. Although the bottom‐up approach is a highly promising method for producing BP for EESD applications, fabricating 2D BP using bottom‐up methods is still a challenge, and related reports are rare. The successful synthesis of other 2D materials, including graphene, silicene, germanene, and stanene, could inspire the preparation of 2D BP through bottom‐up methods.^83^ **Table** **2** summarizes the possible methods that could be used for EESD applications. Given its limited scalability, tape exfoliation severely restricts the usage of 2D BP for EESDS. Similarly, laser irradiation and pulsed laser deposition are generally unsuitable for the massive production of 2D BP for EESDs because of their high cost. Nevertheless, CVD, which is a well‐established material processing technique in the industry, could be a potential and efficient method for producing high‐quality 2D BP.^141^ **Figure** **11** compares six methods of preparing 2D BP, which are categorized by the number of layers of BP. The figure shows that sonication‐assisted liquid‐phase exfoliation and electrochemical exfoliation are promising fabrication approaches to producing 2D BP for EESD application because of their high scalability, low cost, and high production rate.

**Table 2:** Summary of different methods of preparing 2D BP and the corresponding layers

Method	Experiments	Thickness	Ref.
Sonication‐assisted liquid‐phase exfoliation	Exfoliated in NMP for 24 h	3–5 layers	103
Sonication‐assisted liquid‐phase exfoliation	Exfoliated in NMP/NaOH for 4 h	1–7 layers	101
Sonication‐assisted liquid‐phase exfoliation	Exfoliated in ethanol for 43 h	5–30 layers	142
Sonication‐assisted liquid‐phase exfoliation	Exfoliated in CHP	1–5 layers	108
Sonication‐assisted liquid‐phase exfoliation	Exfoliated in DMF for 15 h	<6 layers	110
Sonication‐assisted liquid‐phase exfoliation	Exfoliated in water for 8 h	≈3 layers	117
Sonication‐assisted liquid‐phase exfoliation	Exfoliated in 0.5 mg mL^−1^ aqueous PVP	≈5 layers	124
Sonication‐assisted liquid‐phase exfoliation	Exfoliated in 2% aqueous SDS	1–5 layers	121
Sonication‐assisted liquid‐phase exfoliation	Exfoliated in 1% aqueous TX‐100	≈20 layers	122
Sonication‐assisted liquid‐phase exfoliation	Exfoliated in [Emim][Tf_2_N]	≈3 layers	126
Sonication‐assisted liquid‐phase exfoliation	Exfoliated in [HOEMIM]‐[TfO]	4–10 layers	127
Electrochemical exfoliation	Counter electrode (Pt wire), bulk BP, electrolyte (0.5 m Na_2_SO_4_), voltage (+7 V), current (≈1 mA)	3–15 layers	132
Electrochemical exfoliation	Counter electrode (Pt foil), bulk BP, electrolyte (0.5 m Na_2_SO_4_), voltage (+1 V for 2 min, +3 V), current (0.25 A)	Monolayer or few layers	143
Electrochemical exfoliation	Counter electrode (Pt sheet), bulk BP, electrolyte (Tetrabutylammonium hexafluorophosphate (TBAP) in DMF), voltage (−2.5 to −15 V)	Controlled layers (2–11 layers)	133
Ball milling	LiOH additive, ball milling for 24 h	Few layers	31
Thermal annealing	Sequentially annealed BP flakes in the flow of air and N_2_/H_2_ mixture	7 layers	137
In situ CVD‐type method	Phase transition of red phosphorus under controlled conditions	4 layers	139
Hydrothermal method	Hydrothermal reaction at 200 °C for 16 h using red phosphorus microspheres/NH_4_F solution	≈4 layers	140

## 3 Air Instability

**Figure 12:**
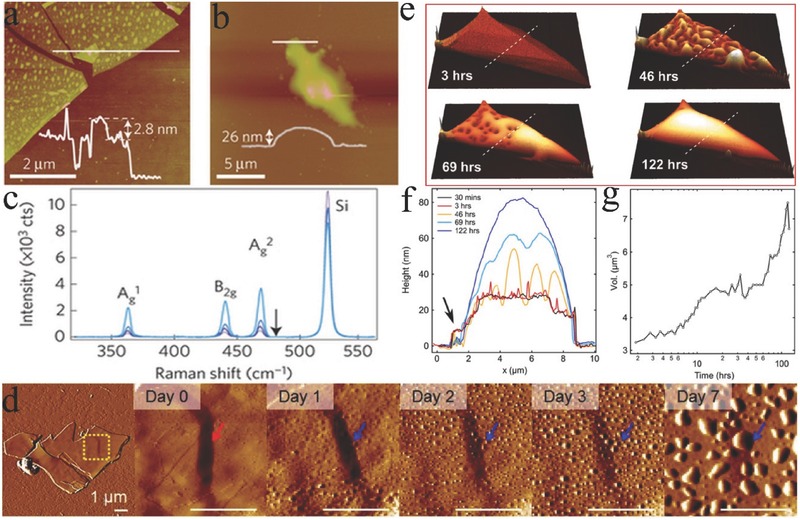
AFM images acquired immediately after mechanical exfoliation on a) a SiO_2_/Si substrate and b) after a few days when the samples were kept in air. Inset: AFM height profiles under the line regions. c) Raman spectra measured in air at 24, 48, 96, and 120 min after mechanical exfoliation. Reproduced with permission.^97^ Copyright 2015, Macmillan Publishers Limited. d) AFM images of BP prepared by mechanical exfoliation. The leftmost image presents the entire flake, and the images progressing to the right present magnified views immediately after exfoliation up to 7 d in ambient conditions. The arrows indicate the same position on the BP flake; all scale bars are 1 µm. Reproduced with permission.^106^ Copyright 2015, American Chemical Society. e) AFM images of a BP flake in air acquired at 3, 46, 69, and 122 h after exfoliation. f) AFM height profiles under the dotted white line regions in panel (e). g) Total volume of the BP flake and water over the test period. Reproduced with permission.^146^ Copyright 2016, IOP publishing.

Unlike most 2D materials studied to date (graphene, TMDs, and h‐BN), which are stable under ambient conditions, BP exhibits air instability.^3^, ^16^ Bulk BP is stable at atmospheric conditions for a few months, but exfoliated BP shows a relatively high reactivity and air instability.^86^ Small bumps can be seen on the surface of the BP flakes (five layers) shortly after exfoliation in ambient conditions (**Figure** **12**a). After a few days, the BP flakes degraded, and large droplets were observed (Figure 12b). The degradation of BP can be quantified by Raman spectroscopy. The Raman peak intensity decreased gradually after continuous exposure in air (Figure 12c).^97^ Similarly, the degradation of BP can be revealed by atomic force microscopy (AFM). Figure 12d shows the AFM images of the mechanically exfoliated BP samples (thicker than 150 nm) after exposure in ambient conditions at different times. No bubbles were observed for the BP samples shortly after sample fabrication. After exposure to ambient conditions for 1 d, bubbles appeared on the BP surface. After 7 d, the bubbles on the samples coarsened to form large bubbles. A theoretical study showed that BP has a strong dipolar moment out of plane, which endows it with a strongly hydrophilic characteristic.^144^ Researchers have attributed the presence of droplets on the surface of the exfoliated BP to adsorbed water.^145^ Island and co‐workers^146^ studied the water condensation on the surface of exfoliated BP (initial thickness from 8 nm at its thinnest part to 30 nm at its thickest part). Water droplets already formed on the surface after 3 h, and water completely covered the flake after 5 d, which in turn led to a large convex meniscus (Figure 12e). The height across the flake is more than doubled over the test period (Figure 12f). The volume increased at a rate of ≈7 µm^3^ min^−1^ in the first 15 h and then increased at a rate of ≈2 µm^3^ min^−1^ after 60 h (Figure 12g). As a result of water absorption, a volume increase of more than 200% was observed after 5 d. The results suggest that the thinner flakes absorbed water faster than the thicker ones, and the appearance of a significant amount of oxygen atom was preferentially localized in the thinnest parts of the BP flake. Marcus–Gerischer theory explains the thickness dependence of exfoliated BP reactivity in terms of electronic confinement.^147^ When a thin sample is synthesized, the band gap shifts toward high energies and close to the energy levels of oxygen acceptor states, thereby strongly enhancing the rate of charge transfer and hence the oxidation rate.^97^ Abellán et al.^148^ also demonstrated that BP degradation is enhanced with the decreasing thickness of the flakes. They also found that lateral dimensions could influence the environmental instability of 2D BP. For example, the environmental degradation of a 2 µm^2^ flake proceeds twice as fast as that of a 7 µm^2^ flake. Absorbed moisture has two adverse effects on 2D BP: (i) physical changes, such as volume expansion and uneven surfaces, and (ii) chemical changes toward a liquid phase, which eventually disappears from the surface.^149^

In most previous studies, researchers simply attributed the degradation of 2D BP in ambient conditions to moisture and strong hydrophilicity.^145^, ^146^, ^150^, ^151^ However, the degradation mechanism of 2D BP is controversial and not entirely clear. Favron et al.^97^ presented a systematic investigation of the degradation mechanisms of BP using AFM, polarized Raman spectroscopy, and TEM combined with high‐angle annular dark‐field (ADF) and hyperspectral electron energy loss spectroscopy. They proved that water, oxygen, and visible light were the three major environmental parameters that were simultaneously required for degrading 2D BP. Their study showed that oxygen and P*_x_*O*_y_* were detected when 2D BP was exposed to air. The light‐induced oxidation reaction was elucidated using the following reaction steps

(1) $$\theta +hv↔\theta^{*}$$

(2) $$\theta^{*}+O_{2(\mathrm{aq})}\to O_{2(\mathrm{aq})}^{\cdot -}+\theta \to \theta_{\mathrm{ox}\cdot }$$

**Figure 13:**
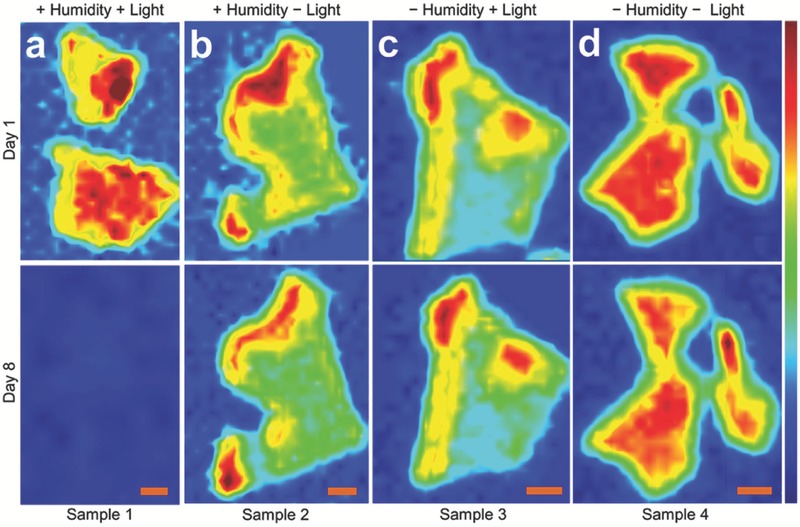
Spatial maps of $A_{g}^{1}$ peak intensity observed on 2D BP crystals stored under different conditions: a) ambient environment, b) absence of light, c) absence of humidity, and d) absence of humidity and light. All scale bars are 2 µm. Reproduced with permission.^154^ Copyright 2016, IOP publishing.

First, 2D BP (θ) was excited by light to generate excited 2D BP (θ*). A charge transfer reaction then occurred between the excited 2D BP and the aqueous oxygen molecules adsorbed on the surface of the 2D BP flake (O_2(aq)_). Finally, the charge transfer reaction with the oxygen–water redox couple produced reactive intermediate species, such as superoxide anions $\left(O_{2(\mathrm{aq})}^{\cdot -}\right)$. The reactive intermediate species reacted with the surface atoms of the 2D BP and etched them into the oxidized species (θ_ox·_). However, Ziletti et al.^152^ believed that the oxidation of 2D BP is possible in pure oxygen atmosphere (under light illumination) without water. In their study, they proposed that O_2_ is initially physisorbed on the surface of 2D BP, and O_2_ is then chemisorbed, leading to the formation of neutral defects and metastable electrically active defect forms, and the dangling oxygen atoms increases the hydrophilicity of 2D BP. However, P–O vibrational modes could be hidden beneath the SiO_2_ substrate vibrational background and could therefore not be detected by Raman spectroscopy. Thus, surface‐enhanced Raman spectroscopy and vibrational sum frequency generation are recommended for the detection of P—O vibrational modes. Nevertheless, Wang et al.^153^ provided an atomic‐level understanding of the stability of 2D BP in terms of its interaction with O_2_ and H_2_O based on the density functional theory (DFT) together with first‐principles molecular dynamics calculations. Their results showed that O_2_ could spontaneously dissociate on BP at room temperature, while H_2_O will not strongly interact with pristine BP. However, an exothermic reaction could occur if BP is first oxidized in air. By contrast, Hanlon et al.^108^ reported that BP can react with water even in the absence of oxygen. Walia et al. ascertained that humidity on its own does not cause any degradation of exfoliated BP.^154^ Nevertheless, three key environmental factors (temperature, humidity, and light) are believed to somehow influence the degradation of BP. Recently, a thorough spectroscopic investigation suggested that temperature has minimal influence on the degradation of BP. **Figure** **13** shows the normalized Raman peak intensity ($A_{g}^{1}$) maps obtained from BP crystals stored under different conditions. Sample 1 was kept in air and exposed to humidity and ambient light. It underwent rapid degradation and deteriorated completely after 8 d (Figure 13a). Sample 2 was stored in an opaque container and exposed to humidity in the absence of light. Only a marginal loss was found after 8 d (Figure 13b). Sample 3 was kept in a desiccator and exposed to light. A slight loss in peak intensity was observed within 8 d, and the lateral dimensions were reduced (Figure 13c). Sample 4 was kept in an opaque container inside a desiccator, which was isolated from humidity and light. No considerable losses occurred (Figure 13d). They concluded that light is the main governing factor of BP degradation and photo‐oxidation originating at the edges of BP crystals. Zhou et al.^155^ proposed a light‐induced ambient degradation process of BP using ab initio electronic structure calculations and molecular dynamic simulation. First, $O_{2}^{-}$ was generated through a charge transfer reaction on the BP surface under ambient light ($O_{2}+hv\overset{P}{\to }O_{2}^{-}+h^{+}$; P and h^+^ refer to 2D BP and a hole, respectively). Then, $O_{2}^{-}$ dissociated on the surface and formed P—O bonds with BP ($O_{2}^{-}$ + P + h^+^ → P*_x_*O*_y_*). Finally, water molecules drew the bonded O out of the surface and removed P from the surface through H‐bond interaction, which resulted in the dissolution of the BP top layer and oxidation of the next layer.

The mechanism responsible for environmental degradation is still a highly debated topic although it has been widely investigated. On the one hand, efficient technologies are still insufficient to confirm BP degradation. For instance, Ziletti et al.^152^ emphasized that conventional Raman spectroscopy was not constantly sensitive to surface‐adsorbed species. On the other hand, the control of thickness or lateral dimensions of 2D BP is still a challenge, in which the two factors could affect the air instability of 2D BP. Well‐defined sample criteria, such as thickness, lateral dimension, and orientation, should be established to quantitatively analyze the degradation process of BP.^148^

## 4 Passivation

Although the exact degradation mechanism of BP has not been explained, the rapid and universal degradation upon exposure to ambient conditions is an invariable issue encountered in 2D BP manipulation. At this point, effective methods should be developed to reduce or eliminate degradation. To date, four strategies, namely, encapsulation, functionalization, liquid‐phase surface passivation, and doping, were actively investigated to passivate the exfoliated 2D BP.

### 4.1 Encapsulation

**Figure 14:**
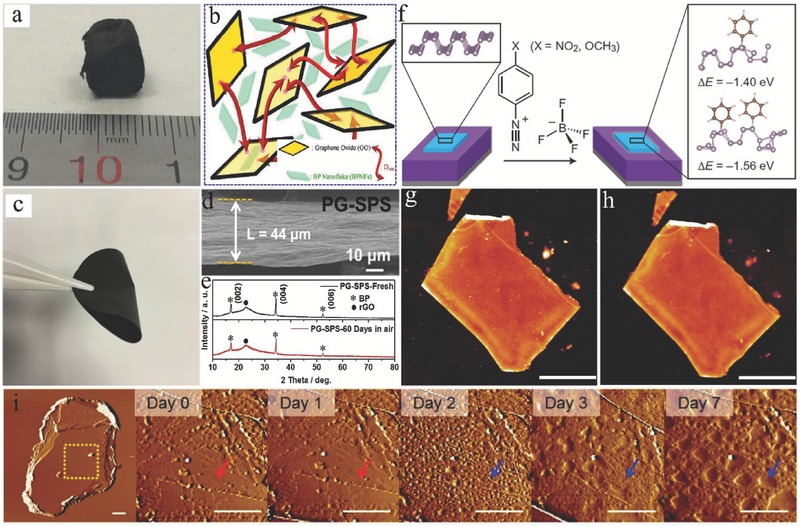
a) Macroscopic view of GO/BPNF aerogel. b) Schematic for GO gelation with the assistance of D_400_ with BPNFs. Reproduced with permission.^166^ Copyright 2017, The Royal Society of Chemistry. c) Photograph of a PGO paper (diameter: 40 mm). d) Cross‐sectional SEM images of PG–SPS paper. e) XRD patterns of fresh PG–SPS paper and PG–SPS paper after exposure to ambience for 60 d. Reproduced with permission.^168^ f) Reaction scheme of aryl diazonium functionalization of BP. AFM images of BP before and after ambient exposure. g) A BP flake immediately after 30 min functionalization in 4‐NBD solution. h) The same flake in panel (b) after 10 d of exposure to ambient conditions. Scale bars are 2 µm. Reproduced with permission.^170^ Copyright 2016, Macmillan Publishers Limited. i) AFM images of BP prepared by solvent exfoliation in NMP. The leftmost image presents the entire flake, and the images progressing to the right present magnified views immediately after exfoliation up to 7 d in ambient conditions. The arrows indicate the same position on the BP flake, and all scale bars are 1 µm. Reproduced with permission.^106^ Copyright 2015, American Chemical Society.

For the encapsulation method, encapsulation layers, such as oxidized aluminum (AlO*_x_*),^149^, ^156^, ^157^, ^158^, ^159^ h‐BN,^151^, ^160^, ^161^ polymer,^100^, ^162^, ^163^, ^164^ SiO_2_,^150^ MoS_2_,^165^ graphene oxide (GO),^166^ and graphene,^161^, ^167^, ^168^ were widely applied as barriers to protect 2D BP from its structure and chemical degradation. AlO*_x_* layer was deposited on BP devices by using the atomic layer deposition (ALD) process, where it can block surface reactions with ambient air. Although ALD AlO*_x_* was an effective and scalable strategy for passivating BP flakes, this process cannot be directly applied to freshly exfoliated 2D BP layers. Similar to ALD AlO*_x_*, h‐BN‐passivation layer was proven effective in preventing the degradation of exfoliated 2D BP. Encapsulation polymer capping, such as poly(methyl methacrylate) and parylene, was also demonstrated to effectively reduce BP degradation. Zhao et al.^164^ demonstrated self‐assembled parylene‐3,4,9,10‐tetracarboxylic dianhydride (PTCDA) monolayers as passivation layers to protect 2D BP. In such passivation, PTCDA molecules did not react with 2D BP and were physically adsorbed on BP surface via weak van der Waals epitaxy, which resulted in excellent air stability. However, all these encapsulation methods were designed only for specialized applications and have sophisticated processes. Zhang and co‐workers^166^ fabricated 3D GO/BP nanoflake hybrid aerogels (GO/BPNFs) by using poly(oxypropylene) diamine (D_400_) as a cross‐linked agent (**Figure** **14**a). First, BPNFs were dispersed within GO nanosheets, where the adjacent GO nanosheets were linked via nucleophilic substitution reactions of epoxide groups in GO with primary amine groups in D_400_.^169^ Thus, BPNFs were fixed within the gallery of GO sheets when the gelation of GO nanosheets occurred (Figure 14b). Zhang et al.^168^ prepared a densely stacked phosphorene–graphene (PG) hybrid paper via spark plasma sintering (SPS) (Figure 14c,d). PG oxide (PGO) paper was first fabricated by simple vacuum filtration and was subjected to SPS to densify and reduce the composite. Excellent air stability of PG paper over a 60 d observation period was achieved considering the effective suppressing permeation of water or oxygen molecules into the deep layers of PG paper, which was confirmed by XRD (Figure 14e).

### 4.2 Functionalization

Recently, the functionalization of 2D BP has been demonstrated as an effective approach to suppress BP degradation. Gao et al.^171^ proposed an approach by combining van der Waals heterostructures using MoSe_2_ as the substrate and a vertical electric field to reverse the stability of physisorption and chemisorption of molecular O_2_ on BP, which significantly enhanced the chemical stability of BP against air oxidation. The enhancement in chemical stability was due to the appropriate manipulation of the relative position of unoccupied orbital of O_2_ molecules in terms of the occupied band of 2D BP. Their approach was supported by first‐principles calculations, and they showed that the lifetime of BP can be extended 10^5^ times using such an approach compared with pristine BP.

Alternatively, chemical functionalization was developed to stabilize the BP structure by the formation of specific bonds between BP and related chemicals. For example, aryl diazonium chemistry was investigated as a method to form covalent P—C bonds with 2D BP by immersing BP samples in aryl diazonium salt solutions (Figure 14f).^170^ After 10 d of exposure to ambient conditions, no visible evidence of oxidation was observed for the functionalized BP (Figure 14g,h). The functionalized 4‐nitrobenzene‐diazonium (4‐NBD)‐modified BP exhibited an apparently improved morphological stability and showed air stability up to 25 d. Similarly, Zhao et al.^172^ demonstrated that titanium sulfonate ligand (TiL_4_) can be used to passivate BP nanosheets to form TiL_4_‐coordinated BP. The finding showed that P—Ti coordination occupied the long‐pair electrons of phosphorus, which prevented the degradation of 2D BP. Recently, Sofer et al.^173^ demonstrated that nucleophilic reagents were highly effective for the formation of P—C and P—O—C bonds in the functionalized BP, which are crucial for improving the chemical stability of 2D BP. Meanwhile, a noncovalent functionalization method was developed to improve the resistance of BP flakes to oxygen degradation.^122^, ^174^ For example, BP was functionalized with parylene diimide (PDI) through van der Waals interactions. The role of PDI was to cover and shield the surface of 2D BP flakes against oxidation. Recently, the surface functionalization of 2D BP was investigated with metallic Ni nanoparticles (NPs). Caporali et al.^175^ demonstrated that Ni NPs immobilized on the surface of exfoliated BP flakes can effectively preserve the morphology of Ni/2D BP flakes for 1 week when kept under ambient conditions in the dark. Meanwhile, metal ions, such as Ag^+^, were used to interact with BP to improve the air stability of BP against oxidation and degradation because cation–π interactions between BP and adsorbed Ag^+^ passivated the long‐pair electrons of P.^176^

### 4.3 Liquid‐Phase Surface Passivation

Among other passivation methods, liquid‐phase (e.g., aqueous solution, organic solvent, and ionic liquid) surface passivation is one of the significant techniques to passivate 2D BP flakes. Notably, exfoliated BP was demonstrated using 1% w/v TX‐100 in water. The TX‐100 head group was able to form a surface‐bound layer, which protected the exfoliated BP from degradation from the surrounding water.^123^ As described in the “Preparation” section, exfoliated BP nanosheets are stable in a number of organic solvents. In a stable organic solvent suspension, BP nanosheets are prevented from reacting with water and oxygen.^108^ Generally, solvent molecules absorbed on BP surface can act as an encapsulation layer to prevent ambient degradation of 2D BP sheets (Figure 14i). Hanlon et al.^108^ demonstrated that solvents with a high boiling point were suitable for passivation. However, the passivation of 2D BP flakes with organic solvents was challenging for potential applications because the protective layer was not easy to remove. As an alternative, ILs are a promising option. Results showed that BP dispersion prepared with ILs can be stabilized in ambient condition for 1 month.^127^ Abellán et al. showed that ILs effectively passivated few layer phosphorene (FLP) flakes of <10 nm for months because ILs were physically bonded on the surface of BP flakes, which provided the BP flakes with outstanding oxidation resistance.^148^ Therefore, large quantities of BP nanosheets with a large area can be fabricated using ILs as exfoliation and surface passivation solvent for a range of applications, especially for energy storage applications.

### 4.4 Doping

Doping was recently demonstrated to be a viable method to improve BP stability. Yang et al. reported that doping with tellurium (Te) enhanced the ambient stability of BP flakes.^177^ No severe corrosion was observed on Te‐doped BP flakes although they were exposed to ambient stability for 4 weeks. On the basis of first‐principles calculations, dopant Te atoms energetically preferred to chemisorb on the surface of BP in a dangling form. According to the calculations, the band edges for the conduction band minimum of Te‐doped BP were close or below the redox potential O_2_/O_2_^−^, whereas the band edges of undoped BP were above the redox potential O_2_/O_2_^−^. This band sheet significantly reduced the generation of light‐induced O_2_.^97^, ^155^ Thus, generating O_2_^−^ anion with the same thickness will be difficult for Te‐doped BP, thereby reducing the oxidization of the Te‐doped BP.

## 5 Electrochemical Energy Storage Application

**Figure 15:**
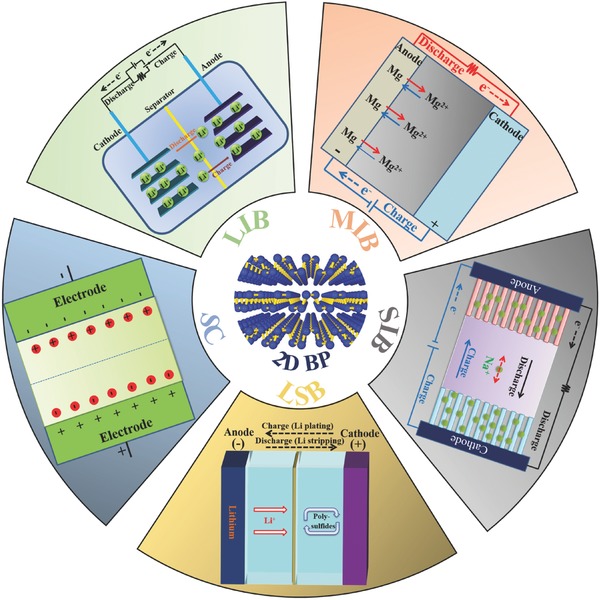
2D BP for electrochemical energy storage.

The fundamental air stability properties of 2D BP make it a strong candidate for numerous applications. Nevertheless, 2D BP is considered an electrochemically active material for the energy storage mechanism (**Figure** **15**), ranging from hosting ions (such as Li^+^, Mg^+^, or Na^+^ in metal‐ion batteries) to accumulating electrostatic charges on the surface (as in electric double‐layer capacitors (EDLCs)), as shown in **Table** **3**.

**Table 3:** Summary of 2D BP‐based materials reported for EESDs

Material	Synthetic method of BP	Application	Electrochemical performance
2D BP–graphene (G) hybrid paper^118^	Exfoliation in deionized water	LIB	The specific capacity is 920 mA h g^−1^ at 100 mA g^−1^ and 141 mA h g^−1^ at 2500 mA g^−1^, and the capacity retention is 80.2% after 500 cycles at 500 mA g^−1^.
2D BP–G Paper^168^	Exfoliation in NMP	LIB	The specific capacity is 1013.3 and 415 mA h g^−1^ at 100 mA g^−1^ and 10 A g^−1^, respectively, and the capacity retention is 91.9% after 800 cycles at 10 A g^−1^.
G‐2D BP/G–G paper^178^	Exfoliation in NMP	LIB	The specific capacity is 1633 and −656 mA h g^−1^ at 100 mA g^−1^ and 1 A g^−1^, respectively, and the capacity retention is 85.8% after 200 cycles at 100 mA g^−1^.
2D BP/Poly(3,4‐ethylenedioxythiophene) (PEDOT)^179^	Exfoliation in DMF	LIB	The specific capacity is 1408 mA h g^−1^ at 100 mA g^−1^ and 434 mA h g^−1^ at 10 A g^−1^, and the capacity retention is 77.6% after 100 cycles at 100 mA g^−1^.
2D BP–carbon nanofiber^104^	Exfoliation in NMP	LSB	1262 mA h g^−1^ at 0.2 C and 785 mA h g^−1^ at 3 C (1 C = 1675 mA g^−1^), and the capacity retention is 73.5% after 500 cycles at 1 C.
2D BP–G^105^	Exfoliation in NMP	SIB	A capacity of 2440 mA h g^−1^ at 0.05 A g^−1^ and 645 mA h g^−1^ at 26 A g^−1^, and 83% capacity retention after 100 cycles at 0.05 A g^−1^.
2D BP^134^	Electrochemical exfoliation	SIB	A capacity of 1878.4 mA h g^−1^ at 100 mA g^−1^ and 321 mA h g^−1^ at 2500 mA g^−1^, and the capacity retention is 102.1% after 100 cycles at 1500 A g^−1^.
2D BP/PEDOT^179^	Exfoliation in DMF	SIB	A capacity of 1397 mA h g^−1^ at 100 mA g^−1^ and 370 mA h g^−1^ at 10 A g^−1^, and 67.4% capacity retention after 100 cycles at 100 mA g^−1^.
2D BP film^112^	Exfoliation in acetone	SC	A capacitance of 17.78 F cm^−3^ at 0.005 V s^−1^ and 1.43 F cm^−3^ at 10 V s^−1^, and a maximum volumetric energy density of 2.47 mW h cm^−3^.
2D BP/G film^78^	Exfoliation in deionized water	SC	A capacitance of 37.5 F cm^−3^ at 0.005 V s^−1^ and 2.42 F cm^−3^ at 1 V s^−1^, and a maximum volumetric energy density of −11.6 mW h cm^−3^.
2D BP/polyaniline^180^	Shear‐force milling	SC	A capacitance of 354 F g^−1^ at 0.3 A g^−1^ and 308 F g^−1^ at 1.7 A g^−1^, and the capacity retention is 96% after 175 cycles at 0.3 A g^−1^.

### 5.1 LIBs

LIBs are one of the important EESDs that can reversibly convert chemical energy into electrical power. They have experienced rapid expansion due to their several important advantages, such as high energy density, low self‐discharging, and no memory effect.^181^, ^182^ In LIBs, Li^+^ ions continuously shuttle between a lithium‐releasing cathode and a lithium‐accepting anode.^183^, ^184^ An LIB generally consists of three components, namely, anode, cathode, and electrolyte. During charging, Li ions are deintercalated from the cathode, passed across the electrolyte, and intercalated in the anode. The process reverses during discharge. The number of ions hosted per gram of material (gravimetric capacity) is crucial to the performance of LIBs.

**Figure 16:**
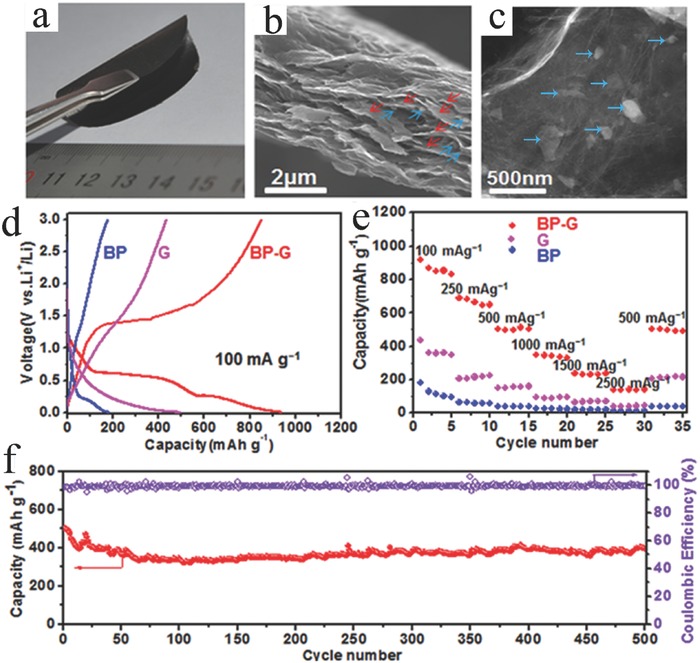
Structure and electrochemical behavior of BP–G hybrid paper. a) Photograph of a BP–G hybrid paper. b) Cross‐sectional view of SEM images of BP–G hybrid paper in panel (a), showing that small BP nanosheets (indicated by blue arrows) are closely contacted with large graphene sheets (indicated by red arrows). c) TEM image of a region of BP–G hybrid paper. d) Second galvanostatic charge/discharge profiles of BP nanosheets, G‐paper, and BP–G hybrid paper electrodes at a current density of 100 mA g^−1^. e) The rate performance of BP nanosheets, graphene paper, and BP–G hybrid paper electrodes at different current densities. f) The cycling stability and Coulombic efficiency (CE) of BP–G hybrid paper electrode at 500 mA g^−1^ for 500 cycles after the rate capability test. Reproduced with permission.^118^

The atomistic lithiation process in BP is similar to that of graphite for LIBs. Li shows a columnar intercalation mechanism and preferentially locates in different 2D BP layers.^185^ Theoretically, 2D BP is a good electrode for high‐capacity LIBs due to the following reasons: (1) Li atoms can bind strongly with phosphorus atoms and exist in a cationic state; (2) the diffusion energy barrier of Li along the ZZ direction is as low as 0.08 eV,^186^ which indicates the possibility of ultrafast charging/discharging; (3) 2D BP can maintain its structure during lithiation and delithiation cycles, and the volume change is only 0.2%;^187^ (4) a large average voltage (2.9 V) can be achieved in 2D BP‐based LIBs;^186^ and (5) the semiconducting‐to‐metallic transition caused by Li intercalation of 2D BP leads to good electrical conductivity, which makes 2D BP an ideal material for LIBs.^187^, ^188^, ^189^ The theoretical specific capacity of monolayer phosphorene is calculated as 432.79 mA h g^−1^.^187^ Zhang et al.^168^ reported the use of FLP prepared by liquid exfoliation as the anode for LIBs, where it exhibited a reversible specific capacity of 210 mA h g^−1^. Coulombic efficiency (CE) was very low (only 11.5%). The inferior performance could be attributed to the severe side reaction from P to Li_3_P (BP → Li*_x_*P → Li_3_P). Therefore, the performance of 2D BP in LIBs should be improved. In accordance with theoretical calculations, Zhang et al.^190^ believed that generating a defect‐free BP for LIB applications was highly important because intrinsic vacancy and the Stone–Wales defect can block lithium migration. Heterostructure design is another important way to improve the performance. As a result of the interfacial synergy effect, 2D BP/graphene (P/G) showed large Li adsorption energy and fast diffusion capability.^191^ The P/G structure also displayed ultrahigh stiffness, which effectively prohibited phosphorene distortion after Li^+^ insertion. This feature was proven by several findings.^118^, ^168^ According to Zhang et al., 2D BP–graphene composite exhibited an improved first‐cycle CE (34.3%) compared with 2D BP (11.5%) and high specific capacity (820 mA h g^−1^) at a current density of 100 mA g^−1^.^168^ Chen et al. demonstrated paper‐like flexible LIB electrodes by combining exfoliated BP nanosheets with graphene sheets (**Figure** **16**a).^118^ The results showed that BP and graphene sheets were contacted closely, and BP sheets were wrapped by large graphene sheets (Figure 16b,c). The 2D BP–graphene composite delivered a specific capacity of 920 mA h g^−1^ at a current density of 100 mA g^−1^ (Figure 16d). The capacity retention was 80.2%, and the average CE approached 100% when cycled at 500 mA g^−1^ over 500 cycles (Figure 16e,f). The good lithium‐storage performance was obtained on a sandwiched thin film, in which two layers of graphene stacks sandwiched a chemically bonded BP/graphene hybrid,^178^ which delivered an initial CE of 71% and 1633 mA h g^−1^ at a current density of 100 mA g^−1^.

Although 2D BP showed significant capability as electrode materials for LIBs, it also experienced several severe drawbacks. For example, individual BP sheets tended to restack because of the van der Waals interaction among them. High SSA of 2D BP resulted in a high initial irreversible capacity and low CE in the first cycle due to the extra electrolyte consumption to generate solid electrolyte interphase layer.^192^ The high SSA also led to undesirable parasitic reactions between the 2D BP and electrolyte, which further resulted in a poor cycle life and potentially caused safety issues.^141^ Generally, the volumetric energy densities of 2D electrodes are low due to the low tap density of 2D electrode materials. The construction of a heterostructure and intercalation can effectively address these issues.^193^, ^194^ Obtaining inspiration from engineering graphene‐based materials is another efficient pathway to acquire high‐performance 2D BP for LIBs.^195^, ^196^, ^197^

### 5.2 LSBs

Early reports on Li–S technology date back to the 1960s. Scientists are currently considering such reports because of the high theoretical specific energy (2600 W h kg^−1^) of such technology, which is more than 5–7 times higher than that of conventional LIBs.^198^, ^199^ Elemental sulfur is highly abundant in nature, nontoxic, and environmentally friendly, thereby making LSBs suitable for commercial applications.^104^ The remarkably high specific energy of LSBs is the direct result of combining two light elements as active materials, namely, metallic lithium anode (theoretical specific capacity: 3860 mA h g^−1^) and elemental sulfur cathode (theoretical specific capacity: 1675 mA h g^−1^).^182^ During discharge, the anodic reaction is the oxidation of lithium, Li → Li^+^ + e^−^, and the cathodic reaction is the reduction of sulfur, S + 2e^−^ → S_2_^−^.^182^ Specifically, elemental sulfur exists in the form of octasulfur; the complete redox reaction of sulfur with lithium can be S_8_ + 16Li → 8Li_2_S.^200^ The complete reaction of sulfur and lithium can lead to an output voltage of 2.2 V per cell.^182^ However, LSBs are affected by several drawbacks, such as (1) low electrical conductivity of sulfur and its various discharge products, (2) large volume changes of sulfur upon cycling, and (3) highly soluble polysulfide intermediates, resulting in a “shuttle effect” during the electrochemical process.^201^ A possible solution to these issues is to encapsulate the sulfur in a suitable matrix to increase electrode conductivity, immobilize soluble polysulfide intermediates, and accommodate sulfur volume changes.

**Figure 17:**
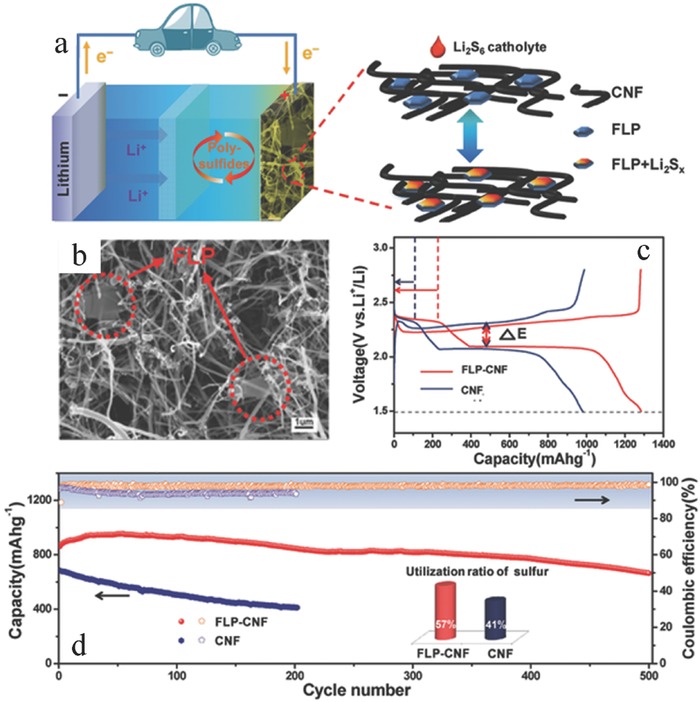
a) Schematic of FLP–carbon nanotube (CNT) matrix used as host for lithium polysulfide catholyte. b) SEM of FLP–CNT. c) Galvanostatic charge–discharge voltage profiles of the first cycle at 0.2 C. d) Cycling stability and CE. The inset shows the utilization of sulfur at a current density of 1 C (calculated based on the maximum capacity during cycling; the theoretical capacity is 1675 mA h g^−1^). Reproduced with permission.^104^

Zhao et al.^202^ first examined the adsorption and diffusion behavior of various polysulfide intermediates at different lithiation/delithiation stations on monolayer BP through theoretical computations. The results showed that the adsorption energy of various polysulfide intermediates to monolayer BP ranged from −1.00 to −2.00 eV, which indicated that monolayer BP was a suitable anchoring material. Considering the charge transfer from intermediate species to 2D BP, the band gaps of hybrid materials became small, which led to improved electrical conductivity. The findings of Li et al. indicated that the introduction of 2D BP reduced the polarization, accelerated the redox reaction, and enhanced the sulfur utilization.^104^ These results implied that 2D BP could be utilized as a suitable anchoring material for LSB cathodes with high performance. A proof‐of‐concept utilizing 2D BP in an LSB with polysulfides is demonstrated in **Figure** **17**a. FLP nanosheets were incorporated into a porous carbon nanofiber (CNF) network (Figure 17b) by simply mixing CNFs with liquid‐exfoliated 2D BP followed by vacuum filtration.^104^ Cyclic voltammetry was used to investigate the electrochemical reaction kinetics. Distinct and stable redox peaks were observed for FLP–CNF electrode. By contrast, the deformed and widened peaks in pure CNF electrode suggested a sluggish kinetic process. The FLP–CNF electrode showed higher reduction and lower oxidation potentials than the pure CNF electrode, thereby suggesting that FLP can significantly reduce electrode polarization. This phenomenon can be attributed to the catalysis effect of FLP on the oxidation/reduction of S/Li_2_S.^203^ A specific capacity of 1262 mA h g^−1^ was obtained at 0.2 C (1 C = 1675 mA g^−1^), whereas the capacity of pure CNF electrode was only 944 mA h g^−1^ (Figure 17c). The high capacity indicated that the polysulfide dissolution was significantly suppressed and polysulfides were confined. With FLP, LSB showed only 0.053% capacity fade per cycle in 500 cycles (Figure 17d), and the average capacity decay per cycle for baseline LSB (without 2D BP) was ≈0.25% over 200 cycles. In addition, the sulfur utilization for FLP–CNF was 57%, which was higher than 41% for pure CNF. The application of 2D BP has achieved significant advancements in LSBs. However, 2D BP‐based LSBs are rarely reported because 2D BP materials are not easy to prepare. The development of 2D BP‐based LSBs still has a long way to go, relying on technological breakthroughs in the efficient fabrication of 2D BP. The Li anode and electrolyte, except the 2D BP‐based cathode, are two other key components for LSBs that require attention.

### 5.3 MIBs

A viable means for postlithium‐ion technology is to replace lithium with magnesium. Metallic magnesium is an ideal anode material for rechargeable batteries due to its advantageous qualities, such as low cost, natural abundance in the Earth's crust,^204^ high melting point (649 °C), a low reduction potential of −2.37 V, and a high volumetric capacity of 3833 mA h cm^−3^.^205^, ^206^ The theoretical specific capacity of magnesium is 2205 mA h g^−1^, and it provides two electrons per atom. The first prototype system for MIBs, which consisted of a metallic magnesium anode, an Mo_6_S_8_ Chevrel‐phase cathode, and an organohaloaluminate salt‐based electrolyte, was presented by Aurbach et al. in 2000.^207^ In this MIB system, magnesium ions deposited/stripped on the anode side and intercalated/deintercalated on the cathode side. Compared with research on LIBs, research on MIBs is still in its initial stages. Several problems hampered the commercialization of MIBs. Electrolyte is the possible difficulty for MIBs. Common magnesium analogs to Li‐ion electrolytes produce passivation layers on the surface of metal anodes. The passivating layers are ionically insulated, and electrochemical activity is inhibited.^208^, ^209^ Although the cathode material in MIBs allowed the insertion of divalent Mg^2+^ ions, the high charge/radius of Mg^2+^ ions caused a strong interaction with ions in the cathode materials, which severely reduced the insertion kinetics of Mg^2+^ ions.^206^ Despite these challenges, MIBs remained attractive candidates for EESDs because of the aforementioned reasons.

Hembram et al.^185^ described the atomistic magnesiation process in BP through first‐principles analysis. The results showed that BP could be utilized as an electrode material for MIBs because it could store magnesium atoms up to Mg_2_P. Using first‐principles calculations, Banerjee and Pati^210^ showed that the synergistic interaction between magnesium ions and covalent P host significantly reduced the magnesium diffusion barrier and optimized the anodic voltage, which could overcome the bottleneck in MIB. Using density functional theory, Jin et al.^211^ investigated the adsorption and diffusion of magnesium atoms on monolayer BP and its structural stability. The adsorption energy was −1.09 eV for magnesium adsorbed on monolayer BP, and the monolayer BP maintained its structural stability in the form of Mg_0.5_P. All theoretical calculations predicted that 2D BP was a promising 2D anode material for MIBs. In the near future, experimental data obtained for MIBs will demonstrate the energy storage potential of 2D BP compared with other nanomaterial‐based MIBs.

### 5.4 SIBs

As alternatives to LIBs, SIBs have attracted significant interest because sodium resources are abundant and inexpensive. In SIBs, aluminum can replace copper as an anodic current collector because sodium does not undergo an alloying reaction with aluminum at a low voltage, which makes SIBs cost effective.^212^ The fundamental principles of SIBs are the same as those of LIBs, that is, sodium ions are shuttled between two electrodes (cathode and anode) through an aqueous/nonaqueous sodium‐ion electrolyte that is contained between the two electrodes on charge and discharge.^213^ The chemical potential difference in the sodium ion between the two electrodes creates a voltage on the battery. However, the larger size of sodium ions (0.106 nm) compared with Li ions (0.076 nm) in ionic radius led to considerable differences in the selected crystal structure and intercalation behavior.^214^ Such differences resulted in slow electrochemical reaction kinetics combined with a large volume change in electrode materials upon the application of charge/discharge current.^215^ Several materials, including layered sodium–transition‐metal oxides, polyanionic compounds, Prussian blue as cathodes and hard carbon, alloys, and low potential transition‐metal oxides and phosphates as anodes, were utilized as electrodes for SIBs.^216^ Although a few recent reports have presented that several cells can compete with LIBs in terms of energy density, the search for appropriate electrode materials for SIBs continues.

**Figure 18:**
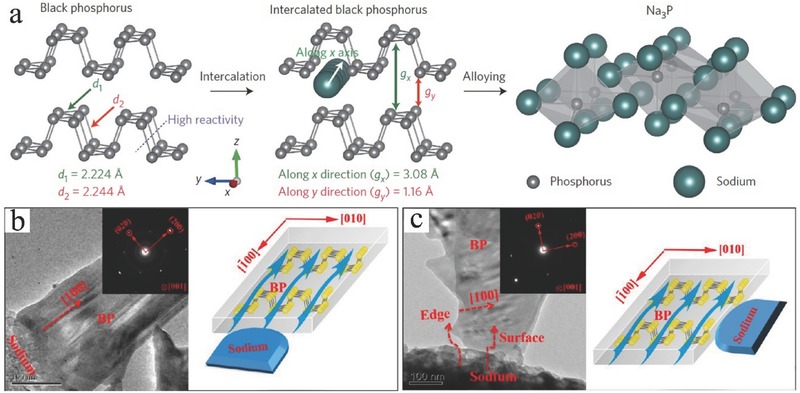
a) Sodiation mechanism in BP. Reproduced with permission.^105^ Copyright 2015, Macmillan Publishers Limited. b) Sodium transport in 2D BP with different contact geometries. Reproduced with permission.^219^ Copyright 2015, American Chemical Society.

Theoretical studies to date have indicated that 2D BP is a promising anode material for SIBs.^185^, ^217^, ^218^ BP has a layered structure similar to that of graphite, but BP has a greater interlayer channel size (3.08 vs 1.86 Å in graphite), which implies that sodium can be stored between the 2D BP layers.^105^ The strong binding energy in sodium–2D BP suggested that sodium can be stabilized on the surface of 2D BP without clustering.^217^ The sodium diffusion on 2D BP is fast and has an energy barrier of only 0.04 eV.^218^ Generally, an intercalation mechanism occurs at low sodium concentration, and the intercalation process then changes to an alloying process at a high sodium content.^185^ Studies have shown that sodium exhibited a planar intercalation mechanism and preferred to localize in the same BP layer.^185^ In addition, the metallic characteristic of sodium–BP at a high sodium concentration provided a significant advantage in electronic conductivity for SIBs.^218^ Moreover, 2D BP exhibited high mechanical stability and integrity upon sodium insertion.^218^ Theoretically, monolayer BP demonstrated exceptionally high theoretical specific capacity due to its ability to adsorb sodium atoms up to the compositions of NaP (865 mA h g^−1^) and Na_2_P (433 mA h g^−1^).^218^ Recently, 2D BP with various layers (from 2 to 11) was investigated as an anode material in SIBs and showed a high capacity of 1968 mA h g^−1^ at a current density of 100 mA g^−1^.^134^ Sun et al.^105^ investigated the sodiation mechanism of BP by using in situ TEM and ex situ XRD techniques. A two‐step sodiation process of intercalation and alloying was demonstrated, which agreed well with theoretical prediction. Sodium ions were first inserted between the 2D BP layers along the *x*‐axis‐oriented channels, and sodiation resulted in the formation of Na*_x_*P species (alloy reaction). The complete sodiation in BP formed Na_3_P (**Figure** **18**a). The transport properties of anisotropic sodium ions within 2D BP were also investigated along in situ aberration‐corrected TEM and DFT simulations, which confirmed the migration of sodium ions along the [100] directions (Figure 18b,c).^219^ Recently, Sun et al. fabricated a 2D BP–graphene sandwich structure via self‐assembly by mixing NMP dispersions of FLP and graphene.^105^ The results showed that a low carbon–phosphorus mole ratio was beneficial to specific capacity because graphene was electrochemically inactive for sodiation. The hybrid material made of FLP sandwiched among graphene layers showed a specific capacity of 2440 mA h g^−1^ (calculated using the mass of BP only) at a current density of 0.05 A g^−1^ and an 83% capacity retention after 100 cycles during cycling between 0 and 1.5 V.

2D BP is a promising anode material for SIBs. However, 2D BP‐based SIBs are still an undeveloped technology for energy storage application. A comprehensive study on the structural and physiochemical properties of BP and their electrochemical properties will lead to significant progress in 2D BP‐based SIBs. The unique 2D structure of BP allows complicated chemical reactions to occur on its surface during charging/discharging. Thus, the interface chemical should be investigated to analyze the nature of Na‐ion cycling.^220^

### 5.5 SCs

**Figure 19:**
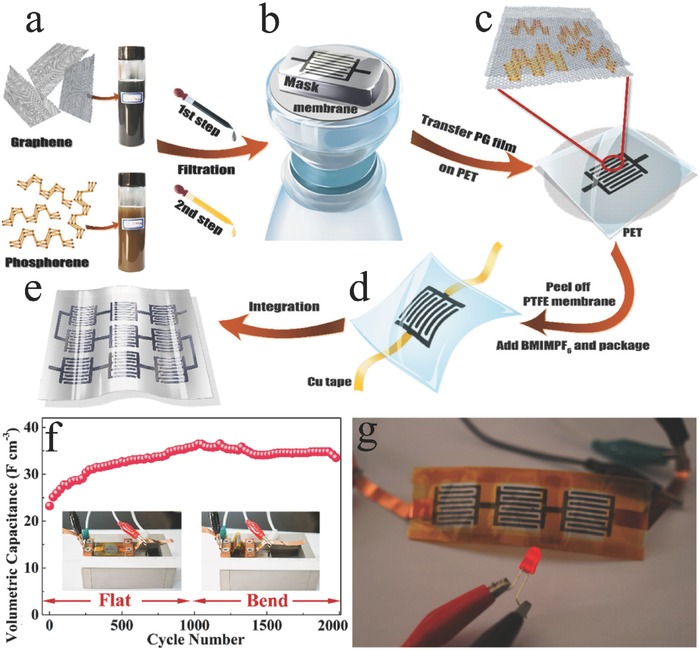
Illustration of the fabrication of PG‐MSCs: a) preparation of graphene and 2D BP inks; b) step‐by‐step filtration of graphene and 2D BP in sequence with the assistance of an interdigital mask; c) dry transfer of PG film onto polyethylene terephthalate (PET); d) peeling off the polytetrafluoroethylene (PTFE) membrane, drop‐casting electrolyte, and device package; e) integration of serially interconnected MSC device. f) Cycling performance of PG‐MSCs obtained at 0.44 A cm^−3^ for 2000 cycles under flat and bent states. The inset is the optical images of PG‐MSCs in flat and bent states. g) Three serial PG‐MSCs can power a red light‐emitting diode. Reproduced with permission.^78^ Copyright 2017, American Chemical Society.

SCs (also called electrochemical capacitors or ultracapacitors) have received increasing research interest in recent years because of their high power density, long cyclic stability, and ultrafast charging–discharging rate. Considering different charge storage mechanisms, SCs can be classified into two types, such as (i) EDLCs and (ii) pseudocapacitors. In EDLCs, energy is stored through electrostatic charge accumulation at the electrode–electrolyte interface, thereby resulting in a double layer. The charges are distributed by physical absorption in EDLCs. Thus, they are not limited by the electrochemical charge transfer kinetics of batteries and can operate at charge–discharge rates in the order of seconds. Carbon materials (e.g., activated carbon) are commonly used as electrodes for EDLCs due to their exceptional properties, such as low electrical resistance, high surface area, chemical inertness, and stability. Pseudocapacitors store energy through fast and reversible surface redox reactions between the electrode active material and electrolyte. Typical pseudocapacitive materials include metal oxides and conducting polymers. These electroactive species enable high energy densities in terms of EDLCs. However, a high energy density is obtained at the expense of low rate capability and reduced cycle life. Surface area and intrinsic conductivity are two critical factors of an electrode material for EDLCs.^138^ The intrinsic properties of 2D BP make it a compelling candidate for SC applications. With the dispersion of exfoliated BP nanoflakes in acetone, Hao et al. demonstrated a facile drop‐casting method to fabricate flexible BP films.^112^ The BP films displayed typical electrochemical double‐layer capacitance behavior. Xiao et al. first demonstrated flexible micro‐SCs based on the interdigital hybrid electrode pattern of 2D BP nanosheets and graphene (PG‐micro‐supercapacitors (MSCs), **Figure** **19**).^78^ PG‐MSCs delivered a maximum volumetric energy density of 11.6 mW h cm^−3^ due to the synergistic effect between 2D BP and graphene. Recently, Sajedi‐Moghaddam et al. investigated a 2D BP/polyaniline (PANI) hybrid material for pseudocapacitors.^180^ The specific capacitance of 2D BP/PANI hybrid reached 354 F g^−1^ at a current density of 0.3 A g^−1^ compared with 308 F g^−1^ of PANI material. The enhanced performance is attributed to the large surface area of 2D BP nanoflakes, which provided a large surface area for the nucleation of PANI and improved the accessibility of ions to PANI active material.

Although 2D BP showed significant potential as an electrode material in SCs, its capacitance should be enhanced for wide and challenging applications. Pore structure, including SSA and pore size distribution, is one of the important factors that significantly affect the performance of an electrode material. The fabrication of porous 2D BP can be an effective approach to improve performance. In addition, doping could be an effective approach to enhance the pseudocapacitance of BP. Accompanied by the aforementioned passivation of BP, doping may lead to a high electrical conductivity and introduce redox capacitance simultaneously. Large SSA, as a 2D material, is frequently lost due to restacking, and the outstanding conductivity would be compromised by absorbed impurities and defects.^221^ In a 3D system, the restacking of 2D BP can be largely avoided, and efficient electrolyte diffusion can be achieved. Meanwhile, rapid and continuous pathways for electronic transport can be provided due to the intimate contact between 2D BP sheets. Thus, the construction of 3D BP electrode architecture is another strategy to improve its performance. Interaction studies between 2D BP and other 2D materials can reveal new opportunities to develop advanced 2D BP‐based electrodes.

## 6 Conclusion and Perspectives

As a new member of the 2D layered material family, monolayer BP and FLP provide many opportunities for investigating fundamental phenomena and practical applications. In this review, we discussed the preparation, structure, and fundamental properties of 2D BP. The use of 2D BP in batteries and SCs is reviewed. High‐quality 2D BP can be prepared successfully through mechanical cleavage, liquid exfoliation, and electrochemical exfoliation. Extensive experimental and theoretical studies demonstrated the outstanding electronic, mechanical, and transport properties of 2D BP; such properties are highly anisotropic and layer dependent. Thus, 2D BP is a promising high‐performance electrode material for most EESDs due to its unique characteristics. Thus far, many significant improvements have been achieved with 2D BP. Despite significant progress in this field, fundamental studies and energy storage application research on 2D BP is still in its early stages, and considerable effort is needed to address unresolved issues and to investigate new concepts.

The first challenge is the production of 2D BP. Considering the unique characteristic of BP, it presents an electrochemical performance that is strongly dependent on its method of production. Tape exfoliation is a simple method and frequently results in monolayer BP or FLP with high quality. However, the production yield of 2D BP by tape exfoliation is low and cannot meet the mass‐scale production required in EESDs. Alternatively, 2D BP can be obtained at a large scale through liquid‐phase exfoliation, including sonication‐assisted liquid‐phase exfoliation and electrochemical exfoliation. However, liquid‐phase exfoliations inevitably introduce intrinsic and extrinsic defects in 2D BP, which adversely affect the electrochemical properties. Thus, feasible techniques should be developed to control defects. As an important bottom‐up method, CVD might be effective in preparing defect‐free 2D BP or 2D BP with controllable defects. However, the production of 2D BP by the CVD method is still challenging due to the absence of a suitable substrate. Other bottom‐up methods, including CVD and hydrothermal synthesis, should be developed. The facile synthesis of 2D BP with the bottom‐up method can draw inspiration from the techniques used for graphene and other 2D materials. Furthermore, the characteristics of 2D BP are highly layer dependent. Thus, the fabrication of 2D BP with tunable layers is significant.

Although most studies on 2D BP were conducted in vacuum or inert gas to test its structure, electrical, and electrochemical properties for EESD applications, air instability under ambient conditions is another significant challenge. The exact chemical reaction mechanism of air instability remains unclear. Generally, four reliable passivation strategies are used, namely, encapsulation, functionalization, liquid‐phase surface passivation, and doping, which were extensively investigated to protect 2D BP from oxygen/moisture degradation. Therefore, the investigation of the mechanism for environment‐induced degradation remains a difficult task. For energy storage applications, graphene is widely used to encapsulate 2D BP, and the integration of 2D BP in a complex architecture or its encapsulation with other electrochemically active nanomaterials is an important direction. The performance can be completely improved simultaneously by passivating the surface of 2D BP. Without altering the properties, functionalization is a viable technology to reduce structural degradation. Solvation shell can protect 2D BP from reacting with water and oxygen in solvent exfoliation. However, related investigations should be conducted. We can investigate new properties and examine energy storage applications for this exciting material by using appropriate passivation process. In addition, doping is a possible approach to preserve the interesting properties of 2D BP.

For 2D BP‐based EESD applications, the conductivity and large surface area of 2D BP are two crucial factors for producing high‐performance devices. In theory, monolayer BP and FLP should have higher electric conductivity due to their 2D structure compared with bulk BP. However, no related data have been reported for 2D BP in terms of theoretical prediction and experimental measurements. The exact values for the surface area of 2D BP are also unknown. In addition, 2D BP should have a larger surface area than graphene does because 2D BP has a puckered structure, whereas graphene has a planar structure. Overall, these fundamental properties require investigation. The utilization of the surface area of 2D BP with high conductivity is a crucial challenge for EESD applications. Constructing a 3D structure is an efficient way to utilize the intriguing properties of 2D BP materials. The 3D hierarchical structure can possess the following advantages: (1) large tunable pore structures that can significantly increase the specific surface area and facilitate ion diffusion kinetics; (2) a high‐electrical‐conductivity 3D network that can provide a fast electron transport pathway; and (3) a 3D‐interconnected structure that can buffer the volume changes during charging/discharging cycling.

The interfacial interactions between 2D BP and other materials play a vital role in enhancing the electrochemical performance of batteries and SCs. However, most studies focus only on the fabrication and characterization of electrodes, and the explanations of the mechanism for the enhancement of electrochemical performance are unclear. An understanding of the atomic‐ and molecular‐level processes that govern the operation, limitations, and failure of various EESDs is the top priority for achieving the full potential of the devices. Current batteries and SC technologies use roll coating method to fabricate electrodes for productions. The synthesis process of 2D BP electrodes is currently limited to research laboratories and is difficult to scale up, thereby hindering the viability of this new material. Therefore, new competitive processing techniques should be developed to revolutionize the development of 2D BP material for energy storage devices. Considering that wearable electronics has become increasingly pervasive in daily life, the demand for flexible energy storage devices is increasing. The utilization of 2D BP in flexible EESDs is an interesting research direction due to the 2D layered structure and flexibility of the material.

The realization of 2D BP is an exciting development that significantly expands the family of 2D materials. Given its intrinsically superior properties, 2D BP is a promising material for EESDs. However, many challenges remain, and the full potential of EESDs built from 2D BP has yet to be realized. Continuous efforts should be made to address the existing issues through theoretical calculations together with experimental investigations. Considerable fundamental and technological breakthroughs can be expected in the coming years.

## Conflict of Interest

The authors declare no conflict of interest.
